# Exploring chemical diversity via a modular reaction pairing strategy

**DOI:** 10.3762/bjoc.8.147

**Published:** 2012-08-15

**Authors:** Joanna K Loh, Sun Young Yoon, Thiwanka B Samarakoon, Alan Rolfe, Patrick Porubsky, Benjamin Neuenswander, Gerald H Lushington, Paul R Hanson

**Affiliations:** 1 Department of Chemistry, The University of Kansas, 1251 Wescoe Hall Drive, Lawrence, KS 66045-7582, USA; 2 The University of Kansas, Center for Chemical Methodologies and Library Development (KU-CMLD), 2034 Becker Drive, Shankel Structural Biology Center, West Campus, Lawrence, KS 66047-3761, USA

**Keywords:** benzoxathiazocine 1,1-dioxides, chemical diversity, informatics, nucleophilic aromatic substitution (S_N_Ar), sultams

## Abstract

The efficient synthesis of an 80-member library of unique benzoxathiazocine 1,1-dioxides by a microwave-assisted, intermolecular nucleophilic aromatic substitution (S_N_Ar) diversification pathway is reported. Eight benzofused sultam cores were generated by means of a sulfonylation/S_N_Ar/Mitsunobu reaction pairing protocol, and subsequently diversified by intermolecular S_N_Ar with ten chiral, non-racemic amine/amino alcohol building blocks. Computational analyses were employed to explore and evaluate the chemical diversity of the library.

## Introduction

The demand for functionally diverse chemical libraries has emerged, as rapid advances in the fields of genomics and proteomics during the “post-genome era” have resulted in an increase in potential therapeutic targets for which there are no known small-molecule modulators [1]. The lack of adequate screening technologies, as well as screening collections of molecules, has hindered these efforts [2–3]. In this regard, recent advances in the construction of chemical libraries that are rich in functional diversity, consisting of appendage, functional group, stereochemical and skeletal diversity, have addressed this challenge and also offer new opportunities [4].

**Figure 1:**
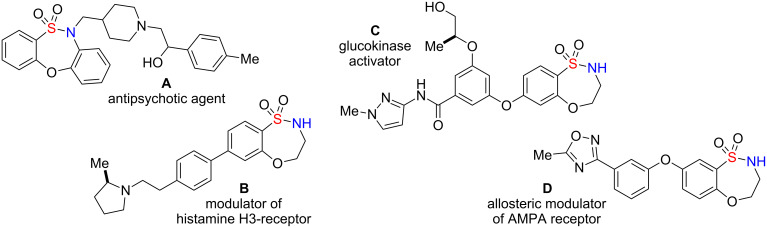
Biologically active benzofused sultams.

**Scheme 1:**
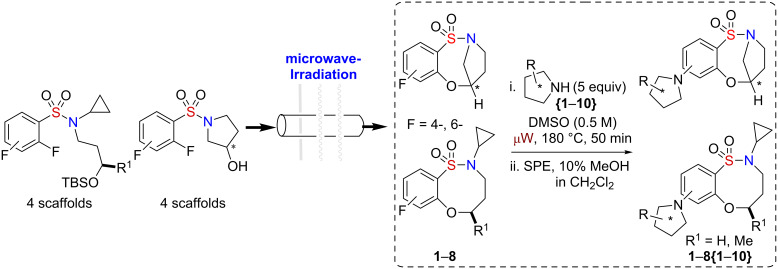
Proposed library generation by microwave-assisted intermolecular S_N_Ar diversification reaction.

Sultams (cyclic sulfonamides) represent a class of compounds with a non-natural chemotype [5–6] that have gained enormous interest in recent years due to their extensive range of biological activities [7–14]. In particular, benzofused sultams, possessing a rich content of sp^3^ amine functionality, have shown a wide biological profile, including antipsychotic activity [15], modulation of histamine H3-receptor [16], glucokinase activation [17–18] and allosteric modulation of AMPA receptor [19], to name but a few (Figure 1). While there are numerous methodologies being reported in the literature for the synthesis of 5-, 6- and 7-membered benzofused sultams, reports on the generation of 8-membered benzofused sultams have been sparse [20–22]. In this regard, our group has focused on the development of several methodologies and protocols for the generation of diverse sultam collections [23–26]. Recent highlights towards these goals include, “click-click-cyclize” [27–28], complementary ambiphile pairing (CAP) [29], and reagent-based DOS [30–31]. In 2011, we reported the development and application of an efficient reaction pairing strategy utilizing three simple reactions, namely sulfonylation, Mitsunobu alkylation and S_N_Ar, which when combined in different sequences or with different coupling reagents, give access to skeletally diverse sultams, including the title compounds and the 8-membered bridged, benzofused sultams [32]. Building on this strategy, we herein report the design and synthesis of an 80-member library of benzofused sultams by a microwave-assisted, intermolecular S_N_Ar diversification of core benzoxathiazocine 1,1-dioxide scaffolds [33–36] (Scheme 1).

## Results and Discussion

**Scheme 2:**
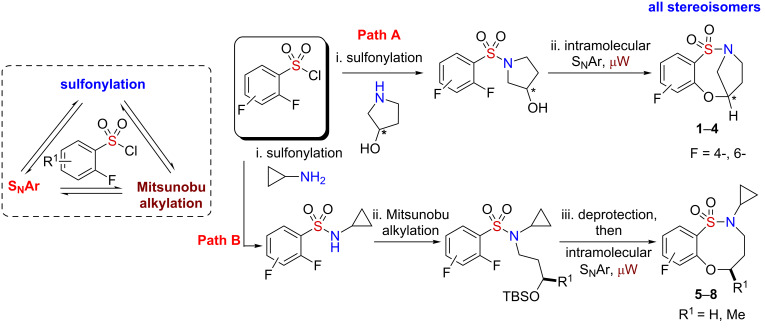
Utilization of a reaction pairing strategy for the synthesis of benzoxathiazocine 1,1-dioxides core scaffolds **1**–**8**.

Initial efforts focused on the synthesis of eight core scaffolds **1**–**8** on multigram scale through the use of three efficient steps, namely sulfonylation, Mitsunobu alkylation and S_N_Ar, to generate both stereoisomers of each core [37] (Scheme 2). The bridged benzofused sultam scaffolds were prepared by a sulfonylation intramolecular S_N_Ar protocol, reported previously [32], utilizing 3-hydroxypyrrolidine in combination with 2,4-difluoro- and 2,6-difluorobenzenesulfonyl chloride. The nonbridged scaffolds were also prepared as reported previously by a sulfonylation intermolecular Mitsunobu alkylation/intramolecular S_N_Ar protocol [32]. 2,4-Difluoro- and 2,6-difluorobenzenesulfonyl chloride were sulfonylated with cyclopropyl amine followed by Mitsunobu alkylation with 3-silyloxybutan-1-ol and subsequent one-pot desilylation intramolecular S_N_Ar alkoxylation (Scheme 2). Each of the scaffolds **1**–**8** was prepared on a 2.5 g scale.

With scaffolds **1–8** in hand, efforts were focused on the diversification of these core scaffolds with a variety of chiral, non-racemic amines/amino alcohols, by intermolecular S_N_Ar utilizing benzoxathiazocine 1,1-dioxide **4** as the test substrate (Table 1). A variety of reaction conditions (equiv of amine, presence of base, concentration of solvent, time and temperature) were examined to identify the optimal conditions. Our initial attempt gave a good yield of 94% with 4.4 equiv of amine, an absence of base, at a concentration of 0.1 M of DMSO, and under microwave irradiation at 150 °C for 20 min (Table 1, entry 2). However, when a hindered amine was utilized it resulted in low (29%) or no yield, even when the reaction time was extended (Table 1, entry 3) or when slightly harsher conditions were used (Table 1, entry 4). Thus, more experiments were performed to investigate other factors, in which the nature and equiv of amine remained the same while the concentration of solvent, temperature and reaction time were increased. Finally, the optimal results were obtained in the absence of base, with 5 equiv of amine, at a concentration of 0.5 M in DMSO, and under 50 min of microwave irradiation at 180 °C (Table 1, entry 10). All reactions were performed under identical conditions, thus attempts were not made to optimize the conditions further for individual substrates.

**Table 1:** Optimization studies for the S_N_Ar reaction utilizing sultam **4**.

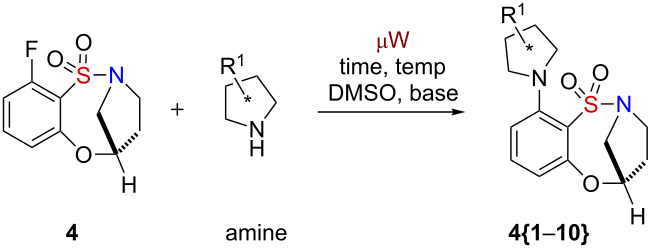							

Entry	Amine	Equiv	Base	Conc. (M)	Time (min)	Temp (°C)	Yield^a^ (%)

1	(*R*)-3-pyrrolidinol	1.3	Cs_2_CO_3_	0.1	30	150	NA
2	(*R*)-3-pyrrolidinol	4.4	—	0.1	20	150	94
3	(*S*)-2-pyrrolidine methanol	4.4	—	0.1	30	150	29
4	(*S*)-2-methoxymethyl pyrrolidine	4.3	—	0.1	50	180	NA
5	(*S*)-3-dimethylamino pyrrolidine	5.0	—	0.1	50	180	88
6	(*R*)-2-methylpyrrolidine	5.0	—	0.1	30	150	42
7	(*R*)-2-methylpyrrolidine	5.0	—	0.1	40	180	62^b^
8	(*R*)-2-methylpyrrolidine	5.0	—	0.1	50	180	70
9	(*R*)-2-methylpyrrolidine	5.0	—	0.1	60	180	35
10	(*R*)-2-methylpyrrolidine	5.0	—	0.5	50	180	95^b^
11	(*R*)-2-methylpyrrolidine	5.0	—	1.0	50	180	83^b^

^a^Yields are reported after flash column chromatography on silica gel. ^b^Crude yield as judged by ^1^H NMR.

### Library design

**Figure 2:**
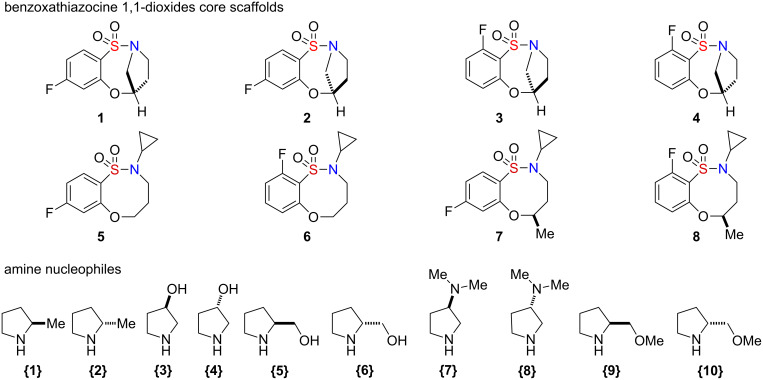
Benzoxathiazocine 1,1-dioxides **1**–**8** and amine library building blocks **{1**–**10}**.

An 80-member, full matrix library was designed by using in silico analysis [38]. Eight benzoxathiazocine 1,1-dioxide scaffolds **1**–**8** were designed, of which library **I** (**1**–**4**) was composed of the entire spectrum of possible stereoisomers, and library **II** (**5**–**8**) was composed of two sets of benzofused sultams having an H or Me group at the R^1^ position. The use of all possible stereoisomers provides the opportunity to generate stereochemical SAR (SSAR) for each building block combination. With the core sultams in hand, a virtual library incorporating all possible combinations of the building blocks of the secondary amines **{1**–**10}** was constructed for each scaffold (Figure 2). Physico-chemical property filters were applied, guiding the elimination of undesirable building blocks that led to products with undesirable in silico properties (see Supporting Information File 1 for full in silico data and detailed information on the calculations). These metric filters included standard Lipinski’s rule of five parameters (molecular weight <500, ClogP <5.0, number of H-acceptors <10, and number of H-donors <5), in addition to consideration of the number of rotatable bonds (<5) and polar surface area. Absorption, distribution, metabolism and excretion (ADME) properties were calculated by using the Volsurf program [39]. Cartesian grid-based chemical diversity analysis was performed according to the method described previously [40], by using standard H-aware 3D BCUT descriptors comparing against the MLSMR screening set (ca. 7/2010; ~330,000 unique chemical structures). Guided by this library design analysis, benzoxathiazocine 1,1-dioxides scaffolds **1**–**8** and amines **{1**–**10}** were chosen to generate the aforementioned 80-member library.

### Validation and library generation

With the optimized conditions in hand, a 20-member validation library was prepared by using scaffolds selected from **1–5** and amines **{1**–**10}** in DMSO (0.5 M) at 180 °C for 50 min, in 1 dram vials, using the Anton Parr Synthos 3000^®^ platform (Table 2) [41]. Upon completion, the crude reaction mixtures were diluted, filtered through silica SPE, and purified by automated mass-directed HPLC. Library validation was essential to assess both substrate and reaction scope, along with evaluating the application of automated mass-directed HPLC as the final analysis and purification method. Key goals for this compound collection were the synthesis of compounds in >90% purity in 40–50 mg quantities, which would be sufficient for HTS screening in the Molecular Library Probe Center Network (MLPCN) (20 mg), for external biological outreach screening partners (20 mg), and to retain a sample (10 mg) for follow-up evaluation or to resupply the NIH MLPCN. Evaluation of this validation library demonstrated that all 20 members were successfully prepared (average purity = 99.7%, yield = 70%, quantity = 73.0 mg) in the desired sultam final masses, with all 20 possessing a final purity >98%.

**Table 2:** Use of a 20-member validation library to probe the reaction scope.

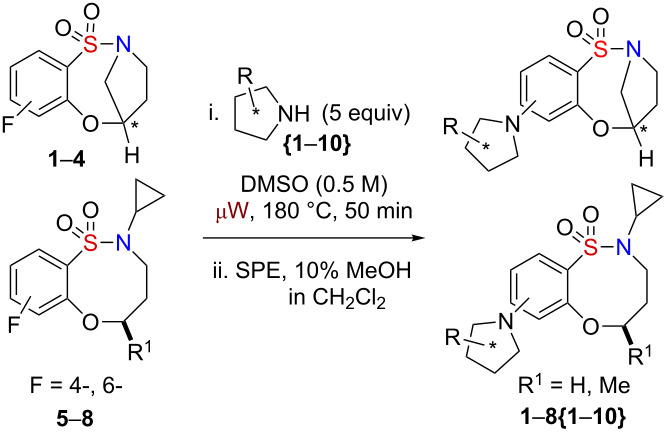							

Sultam^a^	Purity (%)^b^	Yield (%)^b^	Quantity (mg)	Sultam^a^	Purity (%)^b^	Yield (%)^b^	Quantity (mg)

**1{3}**	99.8	78	79.5	**5{1}**	100	80	79.8
**2{3}**	99.4	69	70.0	**5{2}**	100	80	79.4
**3{3}**	100	48	49.3	**5{3}**	100	76	75.8
**4{3}**	99.7	53	54.1	**5{4}**	100	79	79.1
**1{1}**	100	71	71.7	**5{5}**	100	83	85.7
**1{2}**	100	72	73.4	**5{6}**	100	80	83.1
**1{4}**	99.8	75	76.7	**5{7}**	98.2	17	18.7^c^
**1{5}**	99.7	69	73.6	**5{8}**	99.9	46	49.2
**1{6}**	99.6	85	90.2	**5{9}**	99.1	79	85.3
**1{8}**	100	86	94.9	**5{10}**	99.1	78	83.7

^a^Reaction conditions: benzoxathiazocine 1,1-dioxides **1**–**8** (1 equiv, 80 mg), dry DMSO (0.5 M) and amine (5 equiv.); ^b^purified by automated preparative reverse phase HPLC (detected by mass spectroscopy); purity was assessed by HPLC (214 nm); ^c^the low yield obtained was due to instrumental error (see Supporting Information File 1 for more information).

With the validation completed, the remaining 60 compounds of both libraries **I** and **II** were synthesized by the diversification of core benzoxathiazocine 1,1-dioxides scaffolds **1**–**8** and amine **{1**–**10**}. Under the optimal S_N_Ar reaction conditions, libraries **I** and **II** were generated and purified by automated mass-directed HPLC. A total of 80 compounds were prepared and isolated in good yields (average yield 65%), and all compounds had purities greater than 95% after automated purification (see Supporting Information File 1 for all compounds with full numeric data). Final assessment of both libraries **I** and **II** demonstrated that the primary objectives set out in the library design were achieved; final masses ranged between 18–127 mg and the average final mass was 68 mg (original target being 50 mg).

### In silico analysis of chemical diversity and drug-likeness

In silico analysis of the molecular library was performed to achieve enhanced drug-like and lead-like properties, as well as to assess the molecular diversity. In order to assess diversity, five computational analyses were performed, including

1. Cartesian grid-based chemical diversity analysis [40]
2. Overlay analysis
3. Principal moments of inertia (PMI) analysis [42]
4. Conformational analysis
5. Quantitative estimate of drug-like (QED) values [43]

#### Cartesian grid-based chemical diversity analysis

The grid-based diversity analysis protocol, described previously in the Library Design section, provides a simple measure of the relative novelty of a compound. By computing the position of a compound within the molecular property space defined by a large reference set of other interesting compounds, chemical novelty can be estimated from the density of reference compounds in close proximity to the compound of interest. This analysis suggests that our compounds consistently occupy regions of chemical space that are under-represented within the MLSMR reference set. Specifically, all 80 compounds were located in regions with local compound densities of less than the mean value, with compounds **3{3}**, **4{3}**, **4{4}**, **5{3}** and **5{4}** occupying a particularly sparse region of space (all colocating within a cell whose density was 3.5% of the mean density experienced by the reference compounds), while the least unique eight compounds (**5{5}**, **5{6}**, **7{3}**, **7{4}**, **7{5}**, **7{6}**, **8{3}** and **8{4}**) all colocated in a cell with density equal to 78.9% of the mean density experienced by the reference compounds. The mean local density experienced by the 80 compounds reported herein was only 31.7% of the mean density experienced by the reference compounds. All related information can be found in Supporting Information File 1.

#### Overlay analysis

**Figure 3:**
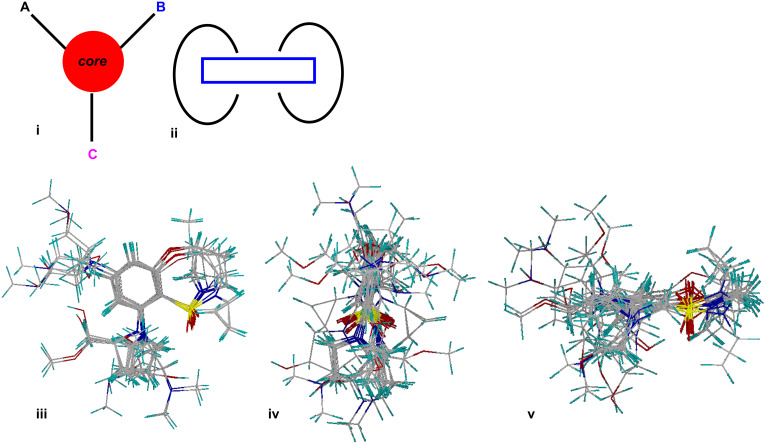
(i) Simple cartoon of the library compounds, with a core of MW ~ 80, based on Lipinski’s rules (MW < 500), and comprising three substituents, each having MW < 140, to establish different functional groups. (ii) This cartoon demonstrates that the substituents extend out of the core in a circular motion. (iii) Overlay images exhibiting the common core in these 80 compounds. (iv) and (v) both overlay images revealing that the substituents are extending outwards in the circular motion as mentioned in (ii).

The overlay produced for the 80 compounds reported herein is depicted in Figure 3 and provides a rudimentary indication of the shape distribution and diversity evident in this library. Orientations 3iv and 3v collectively suggest that the library generally tends toward elongated (rod-like) structures, while the apparent distribution of functional substituents across angles spanning the better part of the whole sphere surrounding the conserved core, suggests that the library as a whole achieves a reasonable level of shape-based diversity.

#### Principal moments of inertia (PMI) analysis

**Figure 4:**
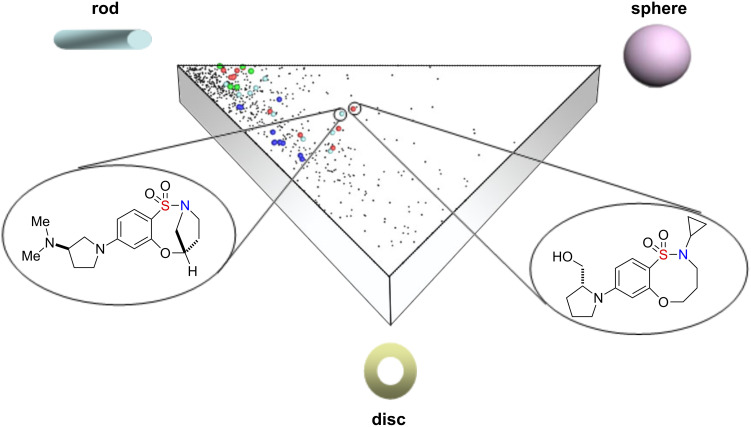
Distribution of 80 compounds (colored spheres) relative to the set of 771 known orally available drugs (black dots) [43].

The rudimentary information gleaned from overlay analysis can be quantified more rigorously via principal moments of inertia (PMI) analysis, which was also employed herein to assess the molecular diversity [42]. PMI analysis utilizes shape-based descriptors: The minimum energy conformation of each library member is determined, PMI ratios are calculated and normalized, and a subsequent triangular plot depicts the shape diversity of the library. The analysis reveals that the 80 compounds generally mirror the shape distribution of the set of 771 known drugs (Figure 4), thus demonstrating the potential drug-likeness of our scaffold. In contrast, some of the compounds are located in the unpopulated region of chemical space, illustrating the novel nature of some of our compounds from the perspective of molecular shape.

#### Conformational analysis

**Figure 5:**
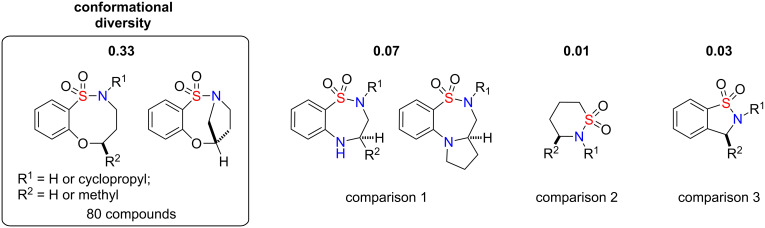
Comparison of a small set of our representative compounds versus two sultams synthesized by our group as well as a biological active compound [11].

While overlay and PMI analysis tend to focus on the shape diversity of libraries as a function of the combined structure of the core scaffold and all known substituents, it is useful to quantify the conformational diversity of the core alone, since this provides additional insight into the prospects for sampling new diversity space as a function of hitherto untested substituents. To quantify this, computations were generated for the mean pairwise atomic root-mean-squared distance (RMSD) using a small set of representative products from the library that was synthesized and compared this value with similar pairwise RMSD calculations for other analogous libraries (Figure 5). In all cases, the structures have been sketched and optimized in SYBYL [44], according to default molecular mechanics settings, and the resulting optimized structures were then all mutually aligned in order to minimize the total pairwise RMSD among conserved scaffold core atoms. The pairwise RMSD values reported in Figure 5 also only correspond to conserved core atoms. The fact that the highlighted core scaffold achieves a much higher RMSD than the other libraries suggests that the scaffold conformation is more sensitive to the choice of substituents, whereas the other libraries exhibit little variation as a function of different substituents. This greater sensitivity on the part of the highlighted library should correspond to greater conformational diversity, which implies sampling of a broader range of property and pharmacophore space than those libraries with lesser conformational diversity.

#### Quantitative estimate of drug-like (QED) values and *Z*-scores

**Figure 6:**
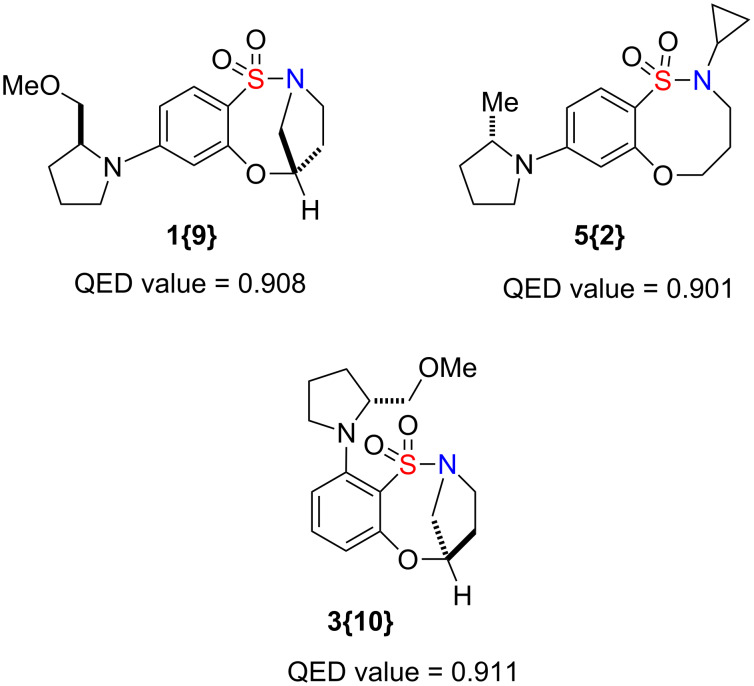
Three representative compounds with high QED values.

While molecular diversity is in itself a topic of intellectual value, in applied sciences it is important to balance this intellectual aspect with suitability toward the intended application. In other words, if one intends to synthesize novel compounds for potential pharmacological applications it is critical that the compounds not only be unique but also be drug-like. Quantifying drug-likeness is one of the numerous methods that are regularly utilized as useful guidelines for early stage drug discovery. A measure of drug-likeness based on the concept of desirability called the quantitative estimate of drug-likeness (QED) has been proposed [43]. The QED concept is a simple approach to multicriteria optimization whereby compound desirability is defined as a function of eight molecular properties, i.e., molecular weight, ALogP, polar surface area, H-bond donor, acceptor, rotatable bond and aryl ring counts, and the presence of structural alerts. The weighted QED values were calculated based on the equation provided by Hopkins et al., mapping compounds to a range from 0 to 1, in which a value of 1 indicates that all properties are within a favorable range. Based on this measure, the 80 compounds reported herein may have elevated prospects for interesting chemical biology: the lowest QED values among these 80 compounds (QED = 0.819 for **1{3}** and **1{4}**) are actually significantly above the mean value (QED = 0.615) for the 771 known drugs analyzed by Hopkins et al., while several distinct scaffolds within our library produced QED values of greater than 0.90 (Figure 6).

**Figure 7:**
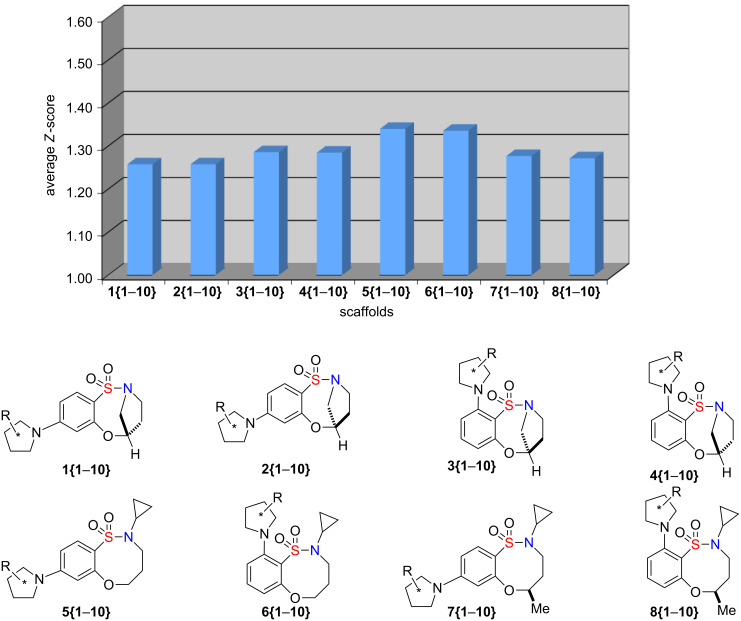
Representation of *Z*-scores for the 80 compounds.

To characterize the QED scores of our scaffolds relative to the reference set of 771 known drugs, we computed mean *Z*-scores for each scaffold and plotted them in Figure 7. Since *Z*-scores of 1.64 and 1.0 correspond to percentile rankings of 95 and 84.1, respectively, it is apparent that all of the reported scaffolds contain compounds with QED values in the upper 80th to lower 90th percentile. The 80 compounds exhibited an average *Z*-score of 1.29, which corresponds to a mean percentile ranking of 90.

## Conclusion

In conclusion, an efficient microwave-assisted intermolecular-S_N_Ar protocol for the synthesis of an 80-member library of amino benzoxathiazocine 1,1-dioxides has been developed. Employing a variety of commercially available chiral, non-racemic amines, the 80-member library of bridged, benzofused, bicyclic sultams was generated by the microwave assisted-S_N_Ar diversification at 4-F and 6-F positions. A series of computational analyses was performed in order to provide pertinent information that guided the second part of the reaction pairing strategy, which will be reported in due course. Further computational analysis revealed that the compounds reported herein generally occupy underrepresented chemical space relative to the MLSMR screening set, but are drug-like both in terms of their distribution in shape space (as compared to a collection of 771 known orally available drugs depicted according to molecular PMI profiles) and according to the QED measure (by which all of this library of compounds are predicted to be significantly more drug-like than the average real drug). Structural overlays and PMI analysis suggest that the highlighted compounds tend to sample a reasonable array of shape space within the range between rod-like and disk-like compounds. RMSD comparisons of a selection of representative structures from this library suggest that the core scaffold has a greater inherent flexibility than comparable products from other related libraries. This flexibility can produce libraries with greater molecular diversity as a function of a fixed number of substituents than is observed for comparably sized libraries arising from more rigid scaffolds. It is our hope that the combination of drug-likeness and inherent molecular diversity evident in this library will produce products that demonstrate interesting behavior in biological screening. To gauge these prospects rigorously, these compounds have been submitted for evaluation of their biological activity in high-throughput screening assays at the NIH MLPCN and the results will be reported in due course.

## Supporting Information

File 1 Experimental procedures, tabulated results for all libraries, and full characterization data for 20 representative compounds.
