# Supplementary material for: Exploring chemical diversity via a modular reaction pairing strategy
Source: Beilstein J Org Chem. 2012 Aug 15;8:1293–302. doi: 10.3762/bjoc.8.147 (PMC3458752; doi:10.3762/bjoc.8.147)
Supplement: File 1 — Experimental procedures, tabulated results for all libraries, and full characterization data for 20 representative compounds. [file Beilstein_J_Org_Chem-08-1293-s001.pdf]

# Supporting Information

# for

# Exploring chemical diversity via a modular reaction pairing strategy

Joanna K. Loh,[1] Sun Young Yoon,[1] Thiwanka B. Samarakoon,[1] Alan Rolfe,[1] Patrick Porubsky,[2] Benjamin Neuenswander,[2] Gerald H. Lushington[1,2] and Paul R. Hanson[1,2]*

Address: [1]Department of Chemistry, The University of Kansas, 1251 Wescoe Hall Drive, Lawrence, KS 66045-7582 and [2]The University of Kansas, Center for Chemical Methodologies and Library Development (KU-CMLD), 2034 Becker Drive, Shankel Structural Biology Center, West Campus, Lawrence, KS 66047-3761

Email: Paul R. Hanson* - phanson@ku.edu

* Corresponding author

## Experimental procedures, tabulated results for all libraries, and full characterization data for 20 representative compounds

# Table of contents


| | |
|---|---|
| General experimental methods | S3 |
| General procedure | S4 |
| Experimental data for compound **5{1}** | S4 |
| Experimental data for compounds **5{3}** and **5{5}** | S5 |
| Experimental data for compound **5{8}** | S6 |
| Experimental data for compounds **5{10}** and **6{4}** | S7 |
| Experimental data for compound **6{6}** | S8 |
| Experimental data for compounds **7{9}** and **8{2}** | S9 |
| Experimental data for compound **8{7}** | S10 |
| Experimental data for compounds **1{2}** and **1{4}** | S11 |
| Experimental data for compounds **1{6}** and **1{9}** | S12 |
| Experimental data for compound **2{3}** | S13 |
| Experimental data for compounds **3{3}** and **4{3}** | S14 |
| Experimental data for compounds **4{1}** and **4{5}** | S15 |
| Experimental data for compound **4{8}** | S16 |
| $^{1}$H NMR and $^{13}$C NMR spectra | S17 |
| Table of mass spectroscopy data, final mass and purity for all library compounds | S37 |
| Lipinski and ADME data | S40 |
| References | S42 |

## General experimental methods

Stirring was achieved with oven-dried, magnetic stir bars. $CH_2Cl_2$ was purified by passage through the Solv-Tek purification system employing activated $Al_2O_3$ [1]. $Et_3N$ was purified by passage through basic alumina and was stored over KOH. Flash column chromatography was performed with $SiO_2$ from Mallinckrodt Chemicals (V120-25, Silica gel, 60 A, 40–63 μm). Thin layer chromatography was performed on silica gel 60 F254 plates (EMD-5715-7, Merck). Deuterated solvents were purchased from Cambridge Isotope Laboratories. $^{1}H$ and $^{13}C$ NMR spectra were recorded on a Bruker Avance spectrometer operating at 400, 500 MHz and 126 MHz, respectively. High-resolution mass spectrometry (HRMS) and FAB spectra were obtained either on a VG Instrument ZAB double-focusing mass spectrometer boron a LCT Premier Spectrometer (Micromass UK Ltd) operating in the ESI mode (MeOH). All library syntheses were carried out in 1 dram vials utilizing a reaction heating block in an Anton Paar® Synthos 3000 synthesizer. Parallel evaporations were performed using a GeneVac EZ-2 Plus evaporator. Automated preparative reverse-phase HPLC purification was performed using a Waters Mass-Directed Fractionation system (Prep Pump 2525, Make-up pump 515, Sample Manager 2767, UV-DAD detection 2996, and Micromass ZD quadrapole mass spectrometer). Purification via preparative chromatography was achieved utilizing a Waters X-Bridge C18 column (19 × 150 mm, 5 μm, w/ 19 × 10 mm guard column) at a flow rate of 20 mL/min. Samples were diluted in DMSO and purified using a elution mixture of water (modified to pH 9.8 by the addition of $NH_4OH$) and $CH_3CN$, running a concentration gradient which increased to 20% $CH_3CN$ over a 4 min period. The corresponding preparative gradient, triggering thresholds, and UV wavelength were selected based on the HPLC analysis of each crude sample. Analytical analysis of each sample after preparative chromatography utilized a Waters Acquity system with UV-detection and mass-detection (Waters LCT Premier). The analytical method conditions included a Waters Aquity BEH C18 column (2.1 × 50 mm, 1.7μm) and elution with a linear gradient of 5% water (modified to pH 9.8 through addition of $NH_4OH$) to 100% $CH_3CN$ at 0.6 mL/min flow rate. Purity of each sample was determined using UV peak area detected at 214 nm wavelength.

Note:

1. **1.** 20 of the 80 compounds were fully characterized with mp, FTIR, $^{1}$H, $^{13}$C NMR and HRMS.
2. **2.** As this is a library synthesis, compounds with a low yield were not resynthesized.

**General procedure: Microwave-assisted $S_NAr$ diversification of benzoxathiazocine-1,1-dioxides 1–8 cores**

Into a 1-dram vial was added benzoxathiazocine-1,1-dioxides **1**–**8** (1 equiv, 80 mg), dry DMSO (0.5 M) and the corresponding amine (5 equiv). The reaction vessel was capped, placed in Anton Paar Synthos 3000® microwave and heated at 180 °C for 50 min [power = 1200 W, 8 min ramp then 50 min hold]. Afterwards, the reaction was diluted with 10% MeOH on $CH_2Cl_2$, filtered through a $SiO_2$ SPE and concentrated. The crude reaction was concentrated and QC/purified by an automated preparative reverse phase HPLC (detected by mass spectroscopy).

**(*R*)-2-Cyclopropyl-8-(2-methylpyrrolidin-1-yl)-2,3,4,5-tetrahydrobenzo[*b*][1,4,5]oxathiazocine 1,1-dioxide 5{1}**

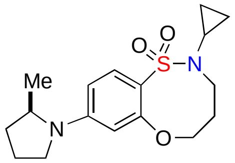


Yellow solid, 79.8 mg (80%).

**mp** 92.5 °C;

**$[\alpha]_D^{20}$** = −66.0° ($c$ = 0.106, $CH_2Cl_2$);

**FTIR** (thin film) 2964, 2930, 1595, 1497, 1464, 1383, 1335, 1202, 1150, 1049 $cm^{-1}$;

**$^{1}$H NMR** (500 MHz, DMSO) δ ppm 7.51 (d, $J$ = 8.8 Hz, 1H), 6.47– 6.34 (m, 2H), 4.27– 4.16 (m, 2H), 3.98– 3.90 (m, 1H), 3.54 (td, $J$ = 5.8, 2.7 Hz, 2H), 3.40 (dd, $J$ = 6.0, 3.8 Hz, 1H), 3.15 (dd, $J$ = 17.7, 8.2 Hz, 1H), 2.10–1.92 (m, 3H), 1.85 (tt, $J$ = 6.9, 3.6 Hz, 1H), 1.74–1.66 (m, 3H), 1.10 (d, $J$ = 6.2 Hz, 3H), 0.74–0.66 (m, 2H), 0.64–0.56 (m, 2H);

**$^{13}$C NMR** (126 MHz, DMSO) δ ppm 157.5, 151.1, 131.1, 117.3, 106.5, 105.4, 74.6, 53.3, 47.6, 46.6, 32.3, 27.6, 22.9, 22.5, 18.4, 7.7, 7.4;

**HRMS** calculated for $C_{17}H_{25}N_2O_3S$ $(M + H)^+$ 337.1586; found 337.1580 (TOF MS $ES^+$).

**(*R*)-2-Cyclopropyl-8-(3-hydroxypyrrolidin-1-yl)-2,3,4,5-tetrahydrobenzo[*b*][1,4,5]oxa-thiazocine 1,1-dioxide 5{3}**

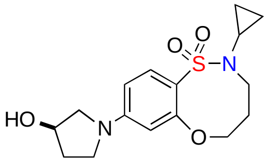


Yellow oil, 75.8 mg (76%).

**$[\alpha]_D^{20}$** = +9.88° (*c* = 0.162, $CH_2Cl_2$);

**FTIR** (neat) 3404, 3394, 2937, 2918, 1597, 1502, 1331, 1254, 1148, 1026 $cm^{-1}$;

**$^1$H NMR** (500 MHz, DMSO) δ ppm 7.52 (d, *J* = 8.8 Hz, 1H), 6.43 (d, *J* = 2.3 Hz, 1H), 6.35 (dd, *J* = 8.8, 2.3 Hz, 1H), 5.03 (d, *J* = 3.7 Hz, 1H), 4.40 (s, 1H), 4.24 (t, *J* = 6.1 Hz, 2H), 3.57–3.51 (m, 2H), 3.43 (dd, *J* = 10.7, 4.6 Hz, 1H), 3.37 (dd, *J* = 9.6, 2.7 Hz, 2H), 3.14 (d, *J* = 10.7 Hz, 1H), 2.03 (dtd, *J* = 13.2, 8.8, 4.6 Hz, 1H), 1.94–1.87 (m, 1H), 1.82 (dt, *J* = 10.4, 3.5 Hz, 1H), 1.73–1.67 (m, 2H), 0.72–0.68 (m, 2H), 0.61 (dd, *J* = 6.7, 2.0 Hz, 2H);

**$^{13}$C NMR** (126 MHz, DMSO) δ ppm 157.4, 151.9, 131.1, 117.5, 106.2, 105.2, 74.5, 69.0, 55.9, 46.7, 45.5, 33.5, 27.6, 22.8, 7.6, 7.5;

**HRMS** calculated for $C_{16}H_{23}N_2O_4S$ $(M + H)^+$ 339.1379; found 339.1339 (TOF MS $ES^+$).

**(*S*)-2-Cyclopropyl-8-(2-(hydroxymethyl)pyrrolidin-1-yl)-2,3,4,5-tetrahydro-benzo[*b*][1,4,5]oxathiazocine1,1-dioxide 5{5}**

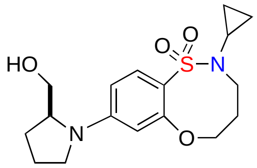


Dark yellow oil, 85.7 mg (83%).

**$[\alpha]_D^{20}$** = −66.4° (*c* = 0.128, $CH_2Cl_2$);

**FTIR** (neat) 3381, 2918, 1595, 1498, 1331, 1153, 1045, 1026 $cm^{-1}$;

**$^1$H NMR** (500 MHz, DMSO) δ ppm 7.51 (d, *J* = 8.8 Hz, 1H), 6.51 (d, *J* = 2.2 Hz, 1H), 6.44 (dd, *J* = 8.9, 2.3 Hz, 1H), 4.83 (dd, *J* = 6.4, 5.3 Hz, 1H), 4.23 (t, *J* = 6.1 Hz, 2H), 3.77 (td, *J* = 7.8, 3.6 Hz, 1H), 3.54 (qt, *J* = 11.7, 5.9 Hz, 2H), 3.48–3.42 (m, 1H), 3.39 (t, *J* = 7.7 Hz, 1H), 3.22 (ddd, *J*

= 10.9, 8.1, 6.7 Hz, 1H), 3.11 (td, $J$ = 9.7, 7.3 Hz, 1H), 2.02–1.83 (m, 5H), 1.75–1.67 (m, 2H), 0.74–0.66 (m, 2H), 0.65–0.57 (m, 2H);

**$^{13}$C NMR** (126 MHz, DMSO) δ ppm 157.4, 151.8, 131.0, 117.8, 106.6, 105.7, 74.6, 60.6, 60.1, 48.1, 46.6, 27.8, 27.6, 22.9, 22.5, 7.7, 7.4;

**HRMS** calculated for $C_{17}H_{25}N_2O_4S$ $(M + H)^+$ 353.1535; found 353.1510 (TOF MS ES$^+$).

**(*S*)-2-Cyclopropyl-8-(3-(dimethylamino)pyrrolidin-1-yl)-2,3,4,5-tetrahydro-benzo[*b*][1,4,5]oxathiazocine 1,1-dioxide 5{8}**

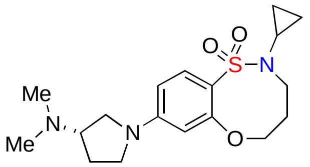


Light brown solid, 49.2 mg (46%).

**mp** 170.5 °C;

**$[\alpha]_D^{20}$** = −32.9° ($c$ = 0.140, $CH_2Cl_2$);

**FTIR** (thin film) 2947, 2866, 2775, 1597, 1501, 1437, 1387, 1333, 1194, 1150, 1074 cm$^{-1}$;

**$^1$H NMR** (500 MHz, DMSO) δ ppm 7.52 (d, $J$ = 8.8 Hz, 1H), 6.47 (d, $J$ = 2.3 Hz, 1H), 6.37 (dd, $J$ = 8.9, 2.3 Hz, 1H), 4.24 (td, $J$ = 6.1, 1.6 Hz, 2H), 3.56–3.48 (m, 3H), 3.46–3.41 (m, 1H), 3.27 (td, $J$ = 10.0, 6.9 Hz, 1H), 3.08–3.03 (m, 1H), 2.77 (dt, $J$ = 15.1, 7.5 Hz, 1H), 2.20 (s, 6H), 2.16 (dd, $J$ = 11.9, 6.4 Hz, 1H), 1.81 (ddd, $J$ = 18.6, 9.2, 6.2 Hz, 2H), 1.71 (ddd, $J$ = 9.8, 6.0, 4.0 Hz, 2H), 0.74–0.66 (m, 2H), 0.61 (d, $J$ = 6.5 Hz, 2H);

**$^{13}$C NMR** (126 MHz, DMSO) δ ppm 157.4, 151.7, 131.0, 117.7, 106.1, 105.3, 74.5, 64.8, 51.9, 46.7, 46.7, 43.9, 40.4, 29.5, 27.6, 22.8, 7.7, 7.4;

**HRMS** calculated for $C_{18}H_{28}N_3O_3S$ $(M + H)^+$ 366.1851; found 366.1826 (TOF MS ES$^+$).

**(*R*)-2-Cyclopropyl-8-(2-(methoxymethyl)pyrrolidin-1-yl)-2,3,4,5-tetrahydro-benzo[*b*][1,4,5]oxathiazocine 1,1-dioxide 5{10}**

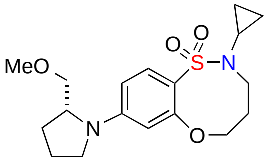


Dark yellow oil, 83.7 mg (78%).

**[α]$_D^{20}$** = +91.9° (*c* = 0.124, $CH_2Cl_2$);

**FTIR** (neat) 2968, 2922, 2878, 1595, 1498, 1435, 1377, 1333, 1177, 1151, 1024 $cm^{-1}$;

**$^1$H NMR** (500 MHz, DMSO) δ ppm 7.52 (d, *J* = 8.8 Hz, 1H), 6.50 (d, *J* = 2.3 Hz, 1H), 6.45 (dd, *J* = 8.9, 2.3 Hz, 1H), 4.23 (td, *J* = 6.2, 2.7 Hz, 2H), 3.98–3.92 (m, 1H), 3.55 (td, *J* = 7.9, 2.8 Hz, 2H), 3.38 (ddd, *J* = 13.7, 13.1, 6.3 Hz, 2H), 3.28–3.24 (m, 4H), 3.13 (dt, *J* = 13.0, 4.8 Hz, 1H), 2.08–1.98 (m, 1H), 1.97–1.90 (m, 3H), 1.88–1.84 (m, 1H), 1.72 (dd, *J* = 9.4, 4.4 Hz, 2H), 0.71 (dd, *J* = 5.8, 2.7 Hz, 2H), 0.62 (dd, *J* = 6.8, 2.4 Hz, 2H);

**$^{13}$C NMR** (126 MHz, DMSO) δ ppm 157.4, 151.7, 131.0, 118.1, 106.7, 105.9, 74.6, 72.0, 58.5, 57.5, 48.1, 46.6, 28.3, 27.6, 22.9, 22.6, 7.8, 7.4;

**HRMS** calculated for $C_{18}H_{27}N_2O_4S$ $(M + H)^+$ 367.1692; found 367.1680 (TOF MS $ES^+$).

**(*S*)-2-Cyclopropyl-10-(3-hydroxypyrrolidin-1-yl)-2,3,4,5-tetrahydrobenzo[*b*][1,4,5]oxa-thiazocine 1,1-dioxide 6{4}**

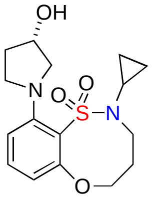


Dark brown oil, 61.0 mg (61%).

**[α]$_D^{20}$** = +266.8° (*c* = 0.196, $CH_2Cl_2$);

**FTIR** (neat) 3385, 2943, 2918, 1589, 1549, 1323, 1236, 1151, 1028 $cm^{-1}$;

**$^1$H NMR** (400 MHz, DMSO) δ ppm 7.22 (dd, *J* = 8.5, 8.0 Hz, 1H), 6.58 (dd, *J* = 10.2, 8.3 Hz, 2H), 4.86 (s, 1H), 4.36 (t, *J* = 6.8 Hz, 2H), 4.25 (d, *J* = 2.7 Hz, 1H), 3.72 (s, 2H), 3.49 (d, *J* = 7.0 Hz, 1H), 3.35 (s, 1H), 3.29 (d, *J* = 4.3 Hz, 1H), 3.05 (d, *J* = 10.9 Hz, 1H), 1.95 (ddd, *J* = 12.5,

11.4, 6.9 Hz, 2H), 1.81 (d, $J$ = 2.8 Hz, 2H), 1.55 (d, $J$ = 3.5 Hz, 1H), 0.73 (s, 1H), 0.52 (ddt, $J$ = 14.0, 11.4, 5.8 Hz, 3H);

**$^{13}$C NMR** (126 MHz, DMSO) δ ppm 159.6, 151.6, 132.0, 115.5, 109.2, 105.2, 70.1, 68.9, 62.0, 54.9, 49.2, 45.8, 33.2, 27.2, 23.1, 5.8;

**HRMS** calculated for $C_{16}H_{23}N_2O_4S$ $(M + H)^+$ 339.1379; found 339.1356 (TOF MS ES$^+$).

**(*R*)-2-Cyclopropyl-10-(2-(hydroxymethyl)pyrrolidin-1-yl)-2,3,4,5-tetrahydro-benzo[*b*][1,4,5]oxathiazocine 1,1-dioxide 6{6}**

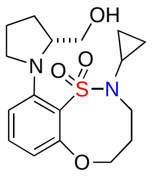


Brown oil, 67.7 mg (65%).

**$[\alpha]_D^{20}$** = +76.0° ($c$ = 0.550, $CH_2Cl_2$);

**FTIR** (neat) 3474, 2945, 2872, 1585, 1474, 1458, 1327, 1237, 1151, 1059 cm$^{-1}$;

**$^1$H NMR** (500 MHz, DMSO) δ ppm 7.32 (t, $J$ = 8.2 Hz, 1H), 6.96 (d, $J$ = 8.4 Hz, 1H), 6.77 (d, $J$ = 7.8 Hz, 1H), 4.42–4.34 (m, 1H), 4.33–4.24 (m, 1H), 4.14 (dd, $J$ = 6.9, 4.8 Hz, 1H), 3.91 (d, $J$ = 3.5 Hz, 1H), 3.87–3.76 (m, 2H), 3.43 (ddd, $J$ = 10.3, 7.0, 3.1 Hz, 1H), 3.32–3.25 (m, 2H), 2.92–2.87 (m, 1H), 2.13–1.98 (m, 2H), 1.84–1.63 (m, 5H), 0.86–0.78 (m, 1H), 0.58–0.48 (m, 3H);

**$^{13}$C NMR** (126 MHz, DMSO) δ ppm 158.4, 151.0, 132.5, 113.7, 110.1, 71.3, 62.4, 60.7, 56.9, 46.7, 28.7, 27.6, 24.6, 23.0, 9.7, 5.1;

**HRMS** calculated for $C_{17}H_{25}N_2O_4S$ $(M + H)^+$ 353.1530; found 353.1519 (TOF MS ES$^+$).

**(*R*)-2-Cyclopropyl-8-((*S*)-2-(methoxymethyl)pyrrolidin-1-yl)-5-methyl-2,3,4,5-tetrahydro-benzo[*b*][1,4,5]oxathiazocine 1,1-dioxide 7{9}**

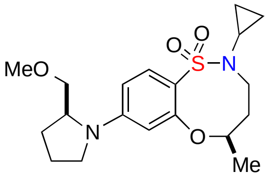


Dark yellow oil, 67.4 mg (63%).

**[α]$_D^{20}$** = −33.0° (*c* = 0.224, $CH_2Cl_2$);

**FTIR** (neat) 2974, 2930, 2878, 1595, 1489, 1435, 1375, 1329, 1209, 1155, 1074 cm$^{-1}$;

**$^1$H NMR** (400 MHz, DMSO) δ ppm 7.52 (d, *J* = 9.0 Hz, 1H), 6.49–6.42 (m, 2H), 4.28 (m, 1H), 3.98–3.86 (m, 2H), 3.43–3.31 (m, 4H), 3.31–3.27 (m, 1H), 3.27–3.19 (m, 2H), 3.18–3.10 (m, 1H), 2.06–1.89 (m, 6H), 1.55–1.46 (m, 1H), 1.37 (d, *J* = 6.4 Hz, 3H), 0.91–0.82 (m, 1H), 0.75–0.66 (m, 1H), 0.64–0.49 (m, 2H);

**$^{13}$C NMR** (126 MHz, DMSO) δ ppm 157.5, 151.5, 130.9, 118.3, 106.8, 106.3, 81.1, 72.2, 58.5, 57.6, 48.1, 43.9, 29.6, 28.3, 27.3, 22.7, 20.9, 9.8, 5.0;

**HRMS** calculated for $C_{19}H_{29}N_2O_4S$ $(M + H)^+$ 381.1848; found 381.1831 (TOF MS ES$^+$).

**(*R*)-2-Cyclopropyl-5-methyl-10-((*S*)-2-methylpyrrolidin-1-yl)-2,3,4,5-tetrahydro-benzo[*b*][1,4,5]oxathiazocine 1,1-dioxide 8{2}**

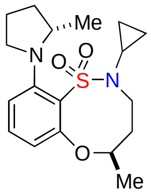


Dark yellow solid, 33.9 mg (35%).

**mp** 111.5 °C;

**[α]$_D^{20}$** = −29.4° (*c* = 0.330, $CH_2Cl_2$);

**FTIR** (thin film) 2968, 2918, 1572, 1454, 1441, 1377, 1319, 1236, 1140, 1055 cm$^{-1}$;

**$^1$H NMR** (500 MHz, DMSO) δ ppm 7.34 (t, *J* = 8.1 Hz, 1H), 7.03 (d, *J* = 8.2 Hz, 1H), 6.68 (d, *J* = 7.7 Hz, 1H), 4.07 (s, 1H), 3.75 (dd, *J* = 16.0, 7.2 Hz, 3H), 3.50 (d, *J* = 14.8 Hz, 1H), 2.89 (td, *J* = 8.5, 4.9 Hz, 1H), 2.61–2.55 (m, 1H), 2.11–2.04 (m, 1H), 1.93 (dtd, *J* = 14.3, 9.6, 4.6 Hz, 1H),

1.85–1.72 (m, 2H), 1.68 (ddd, *J* = 16.5, 8.2, 3.9 Hz, 1H), 1.47 (dq, *J* = 11.8, 8.7 Hz, 1H), 1.31 (d, *J* = 6.2 Hz, 3H), 0.99 (d, *J* = 6.0 Hz, 3H), 0.92 (d, *J* = 9.2 Hz, 1H), 0.72–0.61 (m, 3H);

**$^{13}$C NMR** (126 MHz, DMSO) δ ppm 151.1, 132.6, 117.7, 116.3, 82.4, 56.2, 55.7, 54.9, 46.6, 33.1, 31.6, 27.7, 23.5, 21.2, 18.7, 9.1, 6.2;

**HRMS** calculated for $C_{18}H_{27}N_2O_3S$ $(M + H)^+$ 351.1742; found 351.1700 (TOF MS ES$^+$).

**(*R*)-2-Cyclopropyl-10-((*R*)-3-(dimethylamino)pyrrolidin-1-yl)-5-methyl-2,3,4,5-tetrahydrobenzo[*b*][1,4,5]oxathiazocine 1,1-dioxide 8{7}**

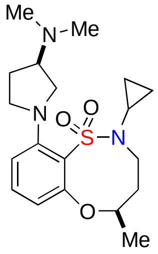


Brown solid, 37.9 mg (36%).

**mp** 91.5 °C;

**$[\alpha]_D^{20}$** = −158.6° (*c* = 0.140, $CH_2Cl_2$);

**FTIR** (thin film) 2972, 2932, 2868, 2824, 1583, 1460, 1377, 1323, 1221, 1155, 1103 cm$^{-1}$;

**$^{1}$H NMR** (400 MHz, DMSO) δ ppm 7.27–7.21 (m, 1H), 6.71 (d, *J* = 8.1 Hz, 1H), 6.54 (d, *J* = 7.2 Hz, 1H), 4.44–4.35 (m, 1H), 3.71 (s, 1H), 3.56 (dd, *J* = 10.4, 6.3 Hz, 2H), 3.42 (dd, *J* = 10.8, 4.7 Hz, 1H), 3.30 (dd, *J* = 9.7, 7.1 Hz, 1H), 3.21 (dd, *J* = 10.4, 5.7 Hz, 1H), 2.70 (m, 1H), 2.12 (s, 6H), 1.97 (td, *J* = 12.6, 6.5 Hz, 3H), 1.83 (td, *J* = 13.7, 7.0 Hz, 1H), 1.71 (d, *J* = 6.9 Hz, 1H), 1.35 (d, *J* = 6.2 Hz, 3H), 0.72 (s, 2H), 0.56 (td, *J* = 6.7, 2.0 Hz, 2H);

**$^{13}$C NMR** (126 MHz, DMSO) δ ppm 158.3, 150.9, 132.4, 119.2, 111.7, 109.4, 79.4, 64.7, 57.2, 54.9, 50.8, 45.4, 43.4, 31.1, 28.9, 27.2, 22.1, 8.2, 6.6;

**HRMS** calculated for $C_{19}H_{30}N_3O_3S$ $(M + H)^+$ 380.2008; found 380.1976 (TOF MS ES$^+$).

**(5*S*)-8-((*S*)-2-Methylpyrrolidin-1-yl)-4,5-dihydro-3*H*-2,5-methano-benzo[*b*][1,4,5]oxathiazocine 1,1-dioxide 1{2}**

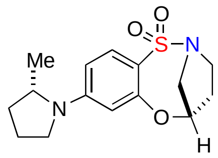

Pale yellow solid, 73.4 mg (72%).

**mp** 143.0 °C;

**[α]$_D$$^{20}$** = +72.3° (*c* = 0.260, $CH_2Cl_2$);

**FTIR** (thin film) 2964, 2918, 2868, 2847, 1599, 1434, 1327, 1142, 1107 $cm^{-1}$;

**$^1$H NMR** (400 MHz, DMSO) δ ppm 7.46 (d, *J* = 8.9 Hz, 1H), 6.40 (dd, *J* = 8.9, 2.4 Hz, 1H), 6.12 (d, *J* = 2.4 Hz, 1H), 5.05 (dd, *J* = 6.4, 2.7 Hz, 1H), 4.01–3.89 (m, 2H), 3.38 (t, *J* = 8.8 Hz, 1H), 3.27 (dd, *J* = 8.3, 3.5 Hz, 1H), 3.20–3.08 (m, 3H), 2.05–1.92 (m, 4H), 1.76 (dt, *J* = 15.4, 7.8 Hz, 1H), 1.70–1.62 (m, 1H), 1.08 (d, *J* = 6.2 Hz, 3H);

**$^{13}$C NMR** (126 MHz, DMSO) δ ppm 152.1, 150.9, 130.8, 116.1, 107.1, 105.4, 80.0, 56.7, 53.3, 47.6, 47.0, 32.3, 28.2, 22.5, 18.4;

**HRMS** calculated for $C_{15}H_{21}N_2O_3S$ $(M + H)^+$ 309.1273; found 309.1243 (TOF MS $ES^+$).

**(5*S*)-8-((*S*)-3-Hydroxypyrrolidin-1-yl)-4,5-dihydro-3*H*-2,5-methano-benzo[*b*][1,4,5]oxathiazocine 1,1-dioxide 1{4}**

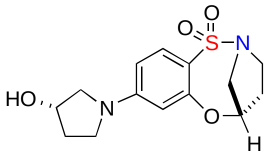

Pale yellow solid, 76.7 mg (75%).

**mp** 208.0 °C;

**[α]$_D$$^{20}$** = +63.1° (*c* = 0.160, $CH_2Cl_2$);

**FTIR** (thin film) 3406, 3009, 2945, 2920, 1601, 1504, 1437, 1321, 1140, 1107 $cm^{-1}$;

**$^1$H NMR** (400 MHz, DMSO) δ ppm 7.47 (d, *J* = 8.9 Hz, 1H), 6.37 (dd, *J* = 8.9, 2.4 Hz, 1H), 6.09 (d, *J* = 2.3 Hz, 1H), 5.05 (dd, *J* = 6.4, 2.7 Hz, 1H), 5.01 (d, *J* = 3.6 Hz, 1H), 4.39 (s, 1H), 3.99 (d,

$J$ = 13.9 Hz, 1H), 3.41 (dd, $J$ = 10.8, 4.6 Hz, 1H), 3.36 (d, $J$ = 6.9 Hz, 1H), 3.32–3.22 (m, 2H), 3.20–3.06 (m, 3H), 2.06–1.94 (m, 2H), 1.93–1.85 (m, 1H), 1.76 (dt, $J$ = 15.3, 6.8 Hz, 1H);

**$^{13}$C NMR** (126 MHz, DMSO) δ ppm 152.1, 151.7, 130.8, 116.3, 106.8, 105.2, 80.0, 69.0, 56.7, 55.9, 47.0, 45.5, 33.4, 28.1;

**HRMS** calculated for $C_{14}H_{19}N_2O_4S$ $(M + H)^+$ 311.1066; found 311.1032 (TOF MS ES$^+$).

**(5*S*)-8-((*R*)-2-(Hydroxymethyl)pyrrolidin-1-yl)-4,5-dihydro-3*H*-2,5-methano-benzo[*b*][1,4,5]oxathiazocine 1,1-dioxide 1{6}**

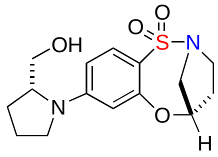


Brown oil, 90.2 mg (85%).

**$[\alpha]_D^{20}$** = +105.5° ($c$ = 0.254, $CH_2Cl_2$);

**FTIR** (neat) 3420, 2972, 2878, 1599, 1497, 1437, 1323, 1140, 1107 cm$^{-1}$;

**$^1$H NMR** (400 MHz, DMSO) δ ppm 7.46 (d, $J$ = 8.9 Hz, 1H), 6.45 (dd, $J$ = 8.9, 2.3 Hz, 1H), 6.20 (d, $J$ = 2.2 Hz, 1H), 5.05 (dd, $J$ = 6.4, 2.6 Hz, 1H), 4.83 (t, $J$ = 5.8 Hz, 1H), 3.99 (d, $J$ = 13.9 Hz, 1H), 3.74 (td, $J$ = 7.8, 3.6 Hz, 1H), 3.41 (ddd, $J$ = 11.7, 9.4, 4.6 Hz, 2H), 3.29–3.05 (m, 5H), 2.05–1.95 (m, 3H), 1.94–1.82 (m, 2H), 1.77 (dt, $J$ = 13.4, 6.8 Hz, 1H);

**$^{13}$C NMR** (126 MHz, DMSO) δ ppm 152.1, 151.6, 130.7, 116.5, 107.3, 105.6, 80.0, 60.5, 60.1, 56.7, 48.1, 47.0, 28.2, 27.7, 22.5;

**HRMS** calculated for $C_{15}H_{21}N_2O_4S$ $(M + H)^+$ 325.1222; found 325.1183 (TOF MS ES$^+$).

**(5*S*)-8-((*S*)-2-(Methoxymethyl)pyrrolidin-1-yl)-4,5-dihydro-3*H*-2,5-methano-benzo[*b*][1,4,5]oxathiazocine 1,1-dioxide 1{9}**

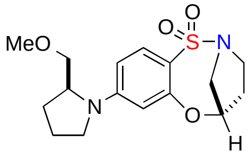


Light brown solid, 84.7 mg (76%).

**mp** 152.0 °C;

$[\alpha]_D^{20} = -36.1°$ ($c$ = 0.180, $CH_2Cl_2$);

**FTIR** (thin film) 2972, 2953, 2928, 2876, 1597, 1495, 1435, 1381, 1327, 1142, 1107 $cm^{-1}$;

**$^1$H NMR** (500 MHz, DMSO) δ ppm 7.47 (d, $J$ = 8.9 Hz, 1H), 6.48 (dd, $J$ = 8.9, 2.3 Hz, 1H), 6.17 (d, $J$ = 2.3 Hz, 1H), 5.06 (dd, $J$ = 6.4, 2.6 Hz, 1H), 4.07–3.95 (m, 1H), 3.93 (d, $J$ = 10.3 Hz, 1H), 3.44–3.34 (m, 3H), 3.30–3.22 (m, 5H), 3.22–3.06 (m, 3H), 2.05–1.88 (m, 4H), 1.78 (dd, $J$ = 14.3, 7.6 Hz, 1H);

**$^{13}$C NMR** (126 MHz, DMSO) δ ppm 152.6, 152.0, 131.2, 117.3, 107.9, 106.2, 80.5, 72.4, 59.0, 58.1, 57.2, 48.5, 47.5, 28.8, 28.7, 23.1;

**HRMS** calculated for $C_{16}H_{23}N_2O_4S$ $(M + H)^+$ 339.1379; found 339.1345 (TOF MS $ES^+$).

**(5*R*)-8-((*R*)-3-Hydroxypyrrolidin-1-yl)-4,5-dihydro-3*H*-2,5-methanobenzo[*b*][1,4,5]oxathiazocine 1,1-dioxide 2{3}**

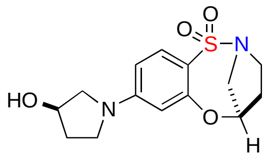


Light yellow solid, 70.0 mg (69%).

**mp** 202.0 °C;

$[\alpha]_D^{20} = -49.2°$ ($c$ = 0.240, $CH_2Cl_2$);

**FTIR** (thin film) 3394, 2949, 2920, 2858, 1601, 1504, 1437, 1321, 1140, 1107 $cm^{-1}$;

**$^1$H NMR** (400 MHz, DMSO) δ ppm 7.47 (d, $J$ = 8.8 Hz, 1H), 6.37 (dd, $J$ = 8.9, 2.4 Hz, 1H), 6.09 (d, $J$ = 2.3 Hz, 1H), 5.05 (dd, $J$ = 6.3, 2.6 Hz, 1H), 5.01 (d, $J$ = 3.6 Hz, 1H), 4.39 (s, 1H), 3.99 (d, $J$ = 13.9 Hz, 1H), 3.41 (dd, $J$ = 10.8, 4.6 Hz, 1H), 3.36 (d, $J$ = 7.0 Hz, 1H), 3.27 (ddd, $J$ = 11.9, 9.8, 4.0 Hz, 2H), 3.18 (dd, $J$ = 13.9, 2.7 Hz, 1H), 3.14–3.06 (m, 2H), 2.06–1.95 (m, 2H), 1.90 (dd, $J$ = 9.7, 6.2 Hz, 1H), 1.81–1.71 (m, 1H);

**$^{13}$C NMR** (126 MHz, DMSO) δ ppm 152.1, 151.7, 130.8, 116.3, 106.8, 105.2, 80.0, 69.0, 56.7, 55.9, 47.0, 45.5, 33.4, 28.1;

**HRMS** calculated for $C_{14}H_{19}N_2O_4S$ $(M + H)^+$ 311.1066; found 311.1014 (TOF MS $ES^+$).

**(5*R*)-10-((*R*)-3-Hydroxypyrrolidin-1-yl)-4,5-dihydro-3*H*-2,5-methanobenzo[*b*][1,4,5]oxathiazocine 1,1-dioxide 3{3}**

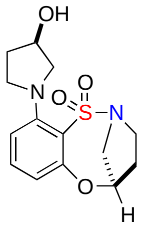

Dark yellow oil, 49.3 mg (48%).

**$[\alpha]_D^{20}$** = +183.3° (*c* = 0.084, $CH_2Cl_2$);

**FTIR** (neat) 3404, 3394, 2962, 2897, 1585, 1464, 1375, 1327, 1140, 1113 $cm^{-1}$;

**$^1$H NMR** (500 MHz, DMSO) δ ppm 7.24 (t, *J* = 8.2 Hz, 1H), 6.72 (d, *J* = 8.6 Hz, 1H), 6.37 (d, *J* = 7.7 Hz, 1H), 5.10 (d, *J* = 4.7 Hz, 1H), 4.86 (d, *J* = 4.1 Hz, 1H), 4.22 (dq, *J* = 12.4, 6.4 Hz, 1H), 3.93 (d, *J* = 13.2 Hz, 1H), 3.51 (td, *J* = 9.5, 6.7 Hz, 1H), 3.42–3.35 (m, 2H), 3.31–3.25 (m, 1H), 3.15 (ddd, *J* = 15.0, 10.6, 4.5 Hz, 1H), 3.06 (t, *J* = 12.1 Hz, 2H), 2.10–2.01 (m, 2H), 1.90 (ddd, *J* = 14.7, 10.5, 5.9 Hz, 1H), 1.73 (dq, *J* = 11.5, 8.1 Hz, 1H);

**$^{13}$C NMR** (126 MHz, DMSO) δ ppm 153.8, 151.0, 132.0, 119.9, 113.3, 112.2, 79.8, 68.9, 59.8, 56.4, 49.7, 46.4, 33.5, 28.5;

**HRMS** calculated for $C_{14}H_{19}N_2O_4S$ $(M + H)^+$ 311.1066; found 311.1028 (TOF MS $ES^+$).

**(5*S*)-10-((*R*)-3-Hydroxypyrrolidin-1-yl)-4,5-dihydro-3*H*-2,5-methanobenzo[*b*][1,4,5]oxathiazocine 1,1-dioxide 4{3}**

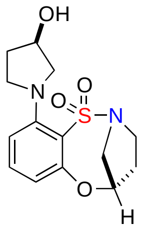

Light brown oil, 54.1 mg (53%).

**$[\alpha]_D^{20}$** = −378.0° (*c* = 2.60, $CH_2Cl_2$);

**FTIR** (neat) 3439, 3418, 1643, 1634, 1589, 1327, 1227, 1138 $cm^{-1}$;

**$^1$H NMR** (500 MHz, $CDCl_3$) δ ppm 7.17 (dd, *J* = 8.4, 7.9 Hz, 1H), 6.71 (dd, *J* = 8.6, 1.0 Hz, 1H), 6.41 (dd, *J* = 7.8, 1.0 Hz, 1H), 4.93 (d, *J* = 4.7 Hz, 1H), 4.47 (s, 1H), 4.37 (d, *J* = 13.2 Hz, 1H),

3.87 (ddd, $J$ = 14.2, 8.4, 5.4 Hz, 2H), 3.28–3.13 (m, 4H), 3.05 (dd, $J$ = 13.2, 1.2 Hz, 1H), 2.37 (s, 1H), 2.29–2.21 (m, 1H), 2.10–2.00 (m, 2H), 1.93–1.84 (m, 1H);

**$^{13}$C NMR** (126 MHz, $CDCl_3$) δ ppm 154.2, 152.0, 132.0, 121.3, 115.3, 113.3, 80.2, 71.1, 61.7, 58.1, 49.6, 46.6, 33.9, 28.8;

**HRMS** calculated for $C_{14}H_{19}N_2O_4S$ $(M + H)^+$ 311.1066; found 311.1051 (TOF MS ES$^+$).

**(5*S*)-10-((*R*)-2-Methylpyrrolidin-1-yl)-4,5-dihydro-3*H*-2,5-methanobenzo[*b*][1,4,5]oxa-thiazocine 1,1-dioxide 4{1}**

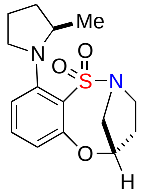


Pale yellow solid, 70.8 mg (70%).

**mp** 139.0 °C;

**$[\alpha]_D^{20}$** = −359.0° ($c$ = 0.286, $CH_2Cl_2$);

**FTIR** (thin film) 2964, 2928, 2889, 2868, 1583, 1460, 1373, 1333, 1227, 1142, 1115 cm$^{-1}$;

**$^1$H NMR** (400 MHz, $CDCl_3$) δ ppm 7.14 (dd, $J$ = 8.5, 7.8 Hz, 1H), 6.72 (d, $J$ = 8.1 Hz, 1H), 6.36 (dd, $J$ = 7.7, 1.0 Hz, 1H), 4.92 (d, $J$ = 4.7 Hz, 1H), 4.42 (dd, $J$ = 13.2, 2.3 Hz, 1H), 4.03–3.91 (m, 2H), 3.32–3.24 (m, 1H), 3.20–3.11 (m, 2H), 3.04 (dd, $J$ = 13.2, 1.2 Hz, 1H), 2.29–2.16 (m, 2H), 1.95–1.81 (m, 2H), 1.72–1.59 (m, 2H), 1.19 (d, $J$ = 6.0 Hz, 3H);

**$^{13}$C NMR** (126 MHz, $CDCl_3$) δ ppm 154.5, 151.6, 131.4, 121.8, 114.6, 113.5, 80.1, 58.4, 55.7, 55.3, 46.4, 34.3, 28.7, 25.3, 19.1;

**HRMS** calculated for $C_{15}H_{21}N_2O_3S$ $(M + H)^+$ 309.1273; found 309.1259 (TOF MS ES$^+$).

**(5*S*)-10-((*S*)-2-(Hydroxymethyl)pyrrolidin-1-yl)-4,5-dihydro-3*H*-2,5-methano-benzo[*b*][1,4,5]oxathiazocine 1,1-dioxide 4{5}**

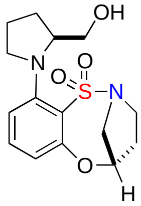

White solid, 72.9 mg (68%).

**mp** 149.0 °C;

**$[\alpha]_D^{20}$** = −128.0° (*c* = 0.530, $CH_2Cl_2$);

**FTIR** (thin film) 3472, 2968, 2874, 1582, 1456, 1331, 1142, 1115, 1067 $cm^{-1}$;

**$^1$H NMR** (400 MHz, $CDCl_3$) δ ppm 7.21 (t, *J* = 8.1 Hz, 1H), 6.95 (d, *J* = 8.1 Hz, 1H), 6.53 (dd, *J* = 7.9, 1.0 Hz, 1H), 4.97 (dd, *J* = 5.1, 1.3 Hz, 1H), 4.37 (d, *J* = 13.5 Hz, 1H), 4.07 (t, *J* = 7.7 Hz, 1H), 3.98 (td, *J* = 9.5, 6.0 Hz, 1H), 3.77 (dd, *J* = 11.3, 2.9 Hz, 1H), 3.42 (t, *J* = 10.0 Hz, 1H), 3.28–3.14 (m, 4H), 3.10 (dd, *J* = 13.5, 1.6 Hz, 1H), 2.26–2.17 (m, 1H), 2.16–2.07 (m, 1H), 2.03 (ddd, *J* = 7.2, 5.6, 2.6 Hz, 1H), 2.00–1.88 (m, 2H), 1.82 – 1.70 (m, 1H);

**$^{13}$C NMR** (126 MHz, $CDCl_3$) δ ppm 153.9, 151.6, 132.1, 125.6, 117.7, 115.9, 80.1, 61.3, 60.7, 57.7, 57.3, 46.7, 29.0, 27.4, 25.7;

**HRMS** calculated for $C_{15}H_{21}N_2O_4S$ $(M + H)^+$ 325.1222; found 325.1194 (TOF MS $ES^+$).

**(5*S*)-10-((*S*)-3-(Dimethylamino)pyrrolidin-1-yl)-4,5-dihydro-3*H*-2,5-methano-benzo[*b*][1,4,5]oxathiazocine 1,1-dioxide 4{8}**

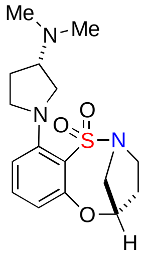


Dark brown oil, 86.3 mg (78%).

**$[\alpha]_D^{20}$** = −449.0° (*c* = 0.152, $CH_2Cl_2$);

**FTIR** (neat) 2955, 1637, 1585, 1464, 1375, 1331, 1223, 1142, 1115 $cm^{-1}$;

**$^1$H NMR** (400 MHz, $CDCl_3$) δ ppm 7.19–7.09 (m, 1H), 6.66 (d, *J* = 8.6 Hz, 1H), 6.40–6.31 (m, 1H), 4.93 (d, *J* = 4.5 Hz, 1H), 4.41 (d, *J* = 13.2 Hz, 1H), 3.88 (td, *J* = 10.8, 6.2 Hz, 1H), 3.58–3.46 (m, 2H), 3.40 (t, *J* = 9.0 Hz, 1H), 3.16 (dd, *J* = 8.8, 6.5 Hz, 2H), 3.04 (d, *J* = 13.1 Hz, 1H), 2.74 (tt, *J* = 10.1, 6.1 Hz, 1H), 2.31 (s, 6H), 2.28–2.21 (m, 1H), 2.20–2.11 (m, 1H), 1.91–1.72 (m, 2H);

**$^{13}$C NMR** (126 MHz, $CDCl_3$) δ ppm 154.5, 151.9, 131.6, 120.0, 114.4, 112.1, 80.2, 65.5, 58.4, 57.2, 52.3, 46.5, 44.6, 31.0, 29.7, 28.7;

**HRMS** calculated for $C_{16}H_{24}N_3O_3S$ $(M + H)^+$ 338.1538; found 338.1517 (TOF MS $ES^+$).

**5{1}**

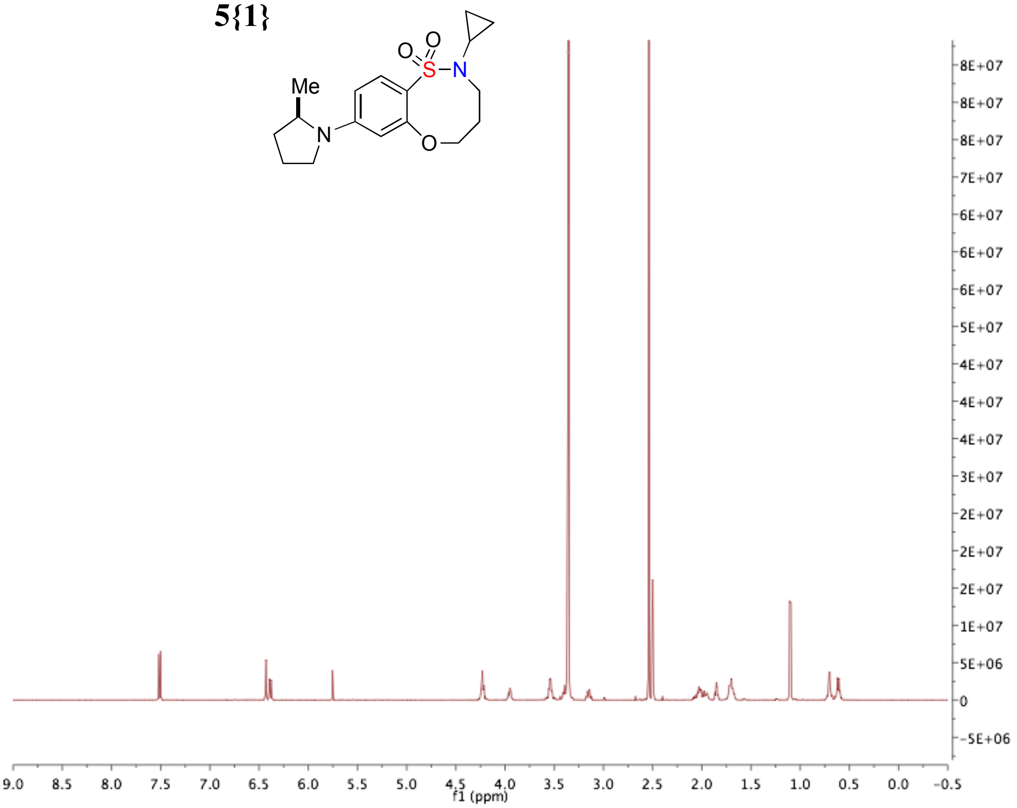


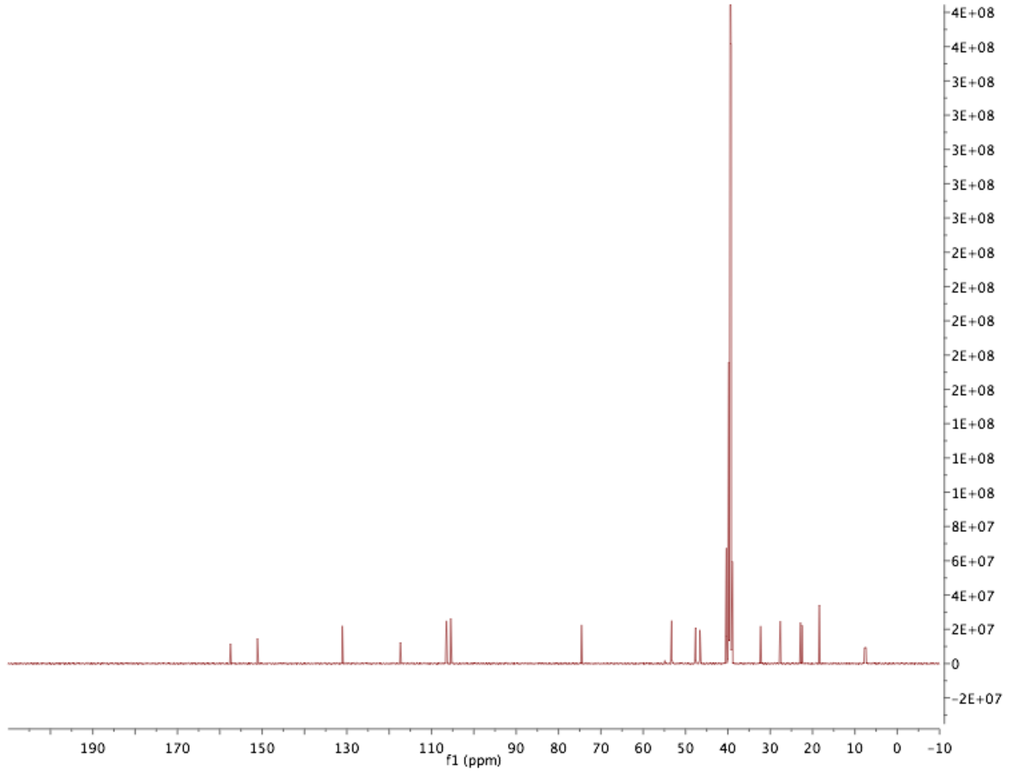

**5{3}**

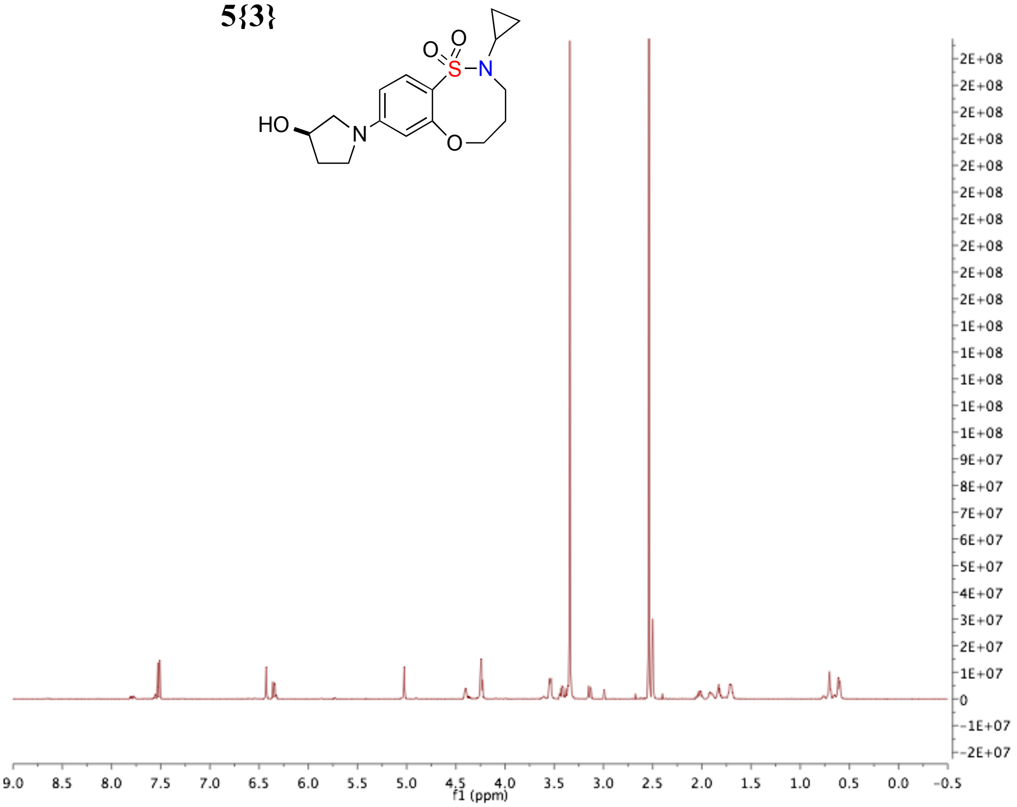


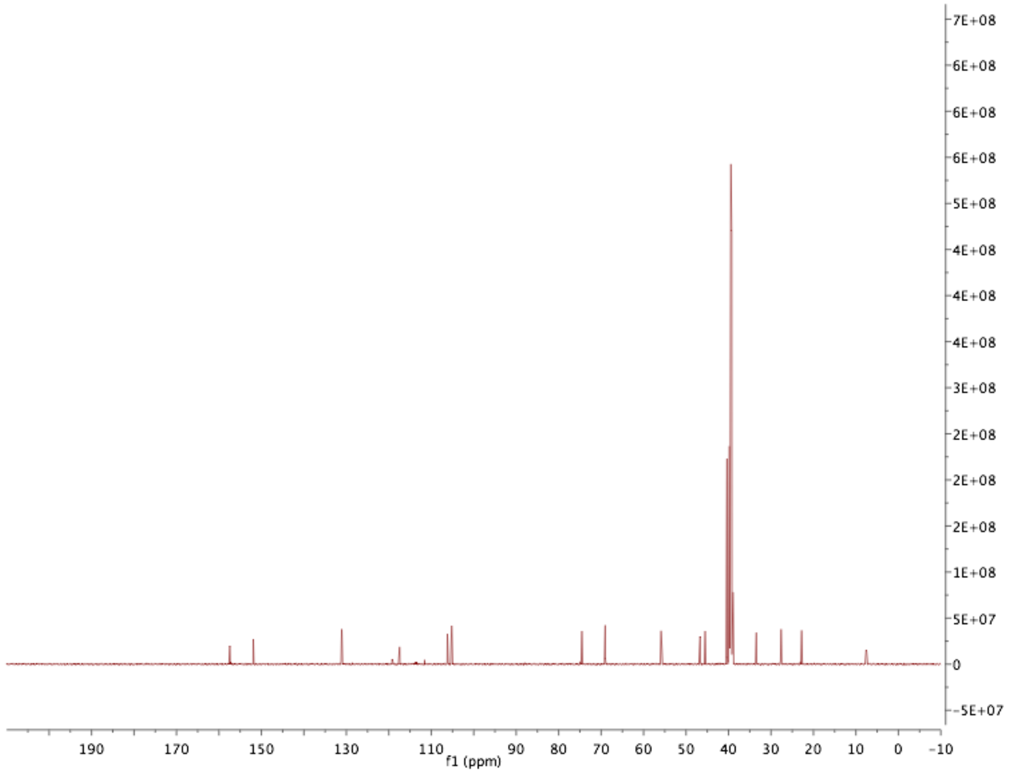

**5{5}**

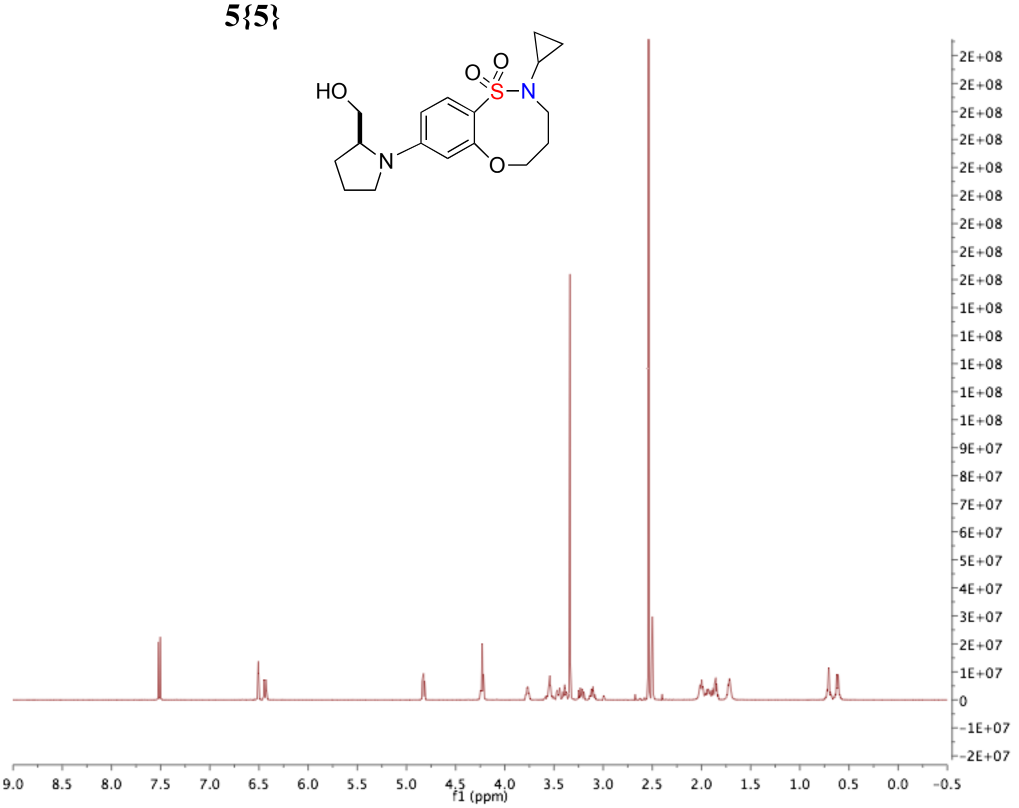

HO
N
O
S
N
O
O
2E+08
1E+08
9E+07
8E+07
7E+07
6E+07
5E+07
4E+07
3E+07
2E+07
1E+07
0
-1E+07
-2E+07
9.0 8.5 8.0 7.5 7.0 6.5 6.0 5.5 5.0 4.5 4.0 3.5 3.0 2.5 2.0 1.5 1.0 0.5 0.0 -0.5
f1 (ppm)


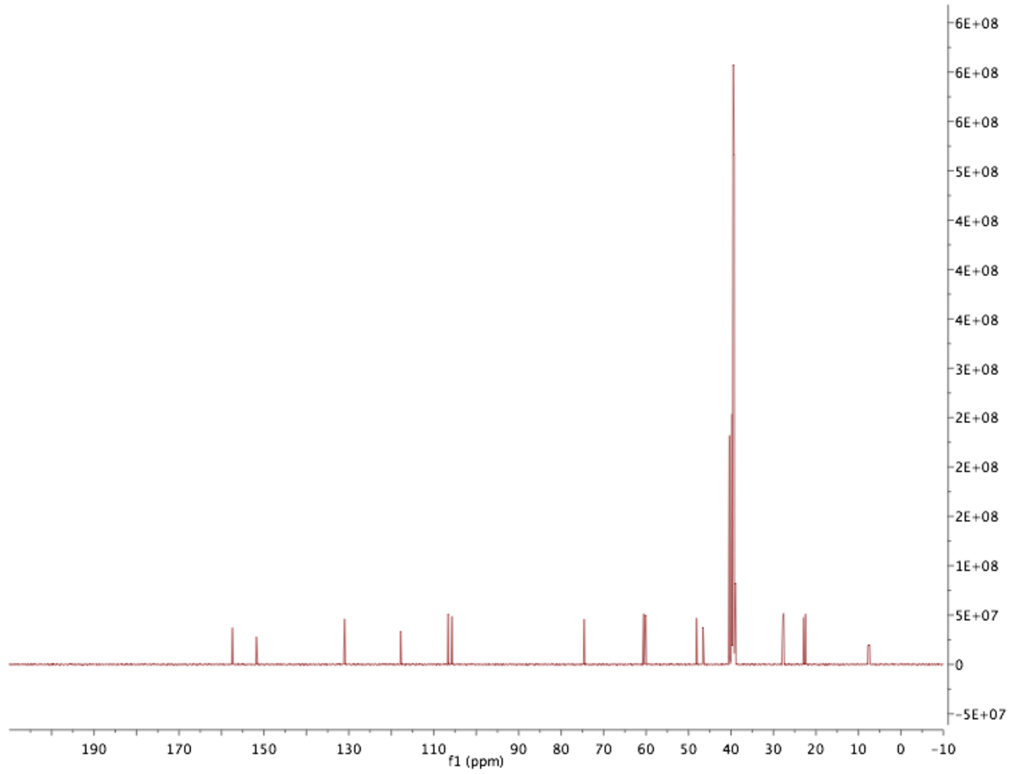

6E+08
5E+08
4E+08
3E+08
2E+08
1E+08
5E+07
0
-5E+07
190 170 150 130 110 90 80 70 60 50 40 30 20 10 0 -10
f1 (ppm)

**5{8}**

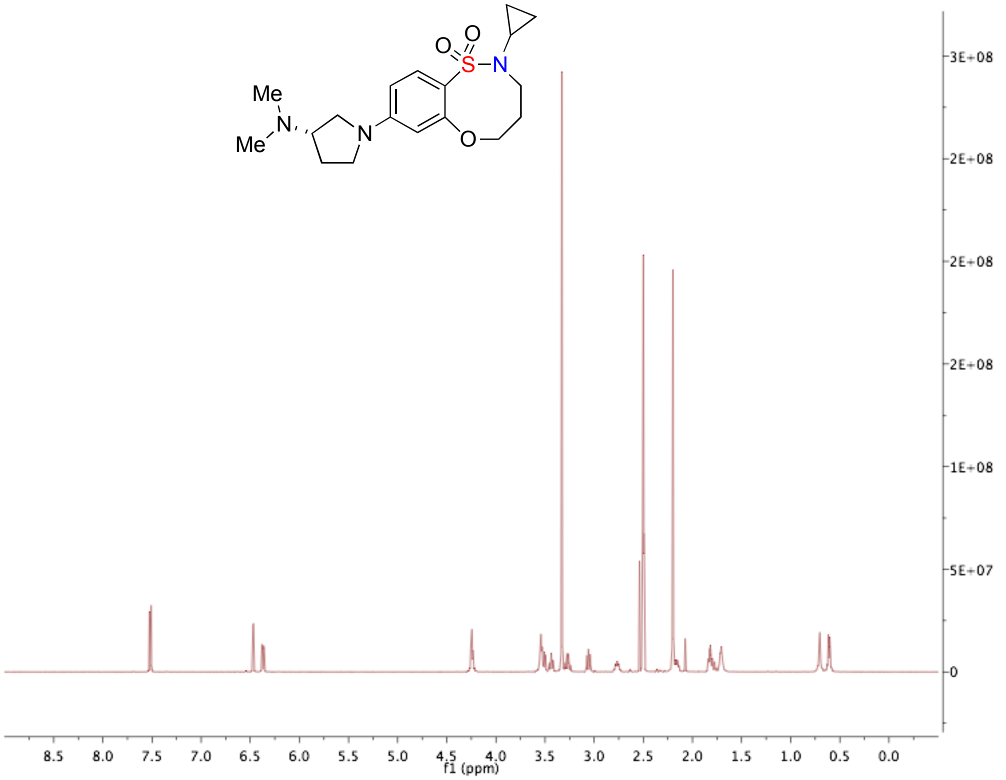


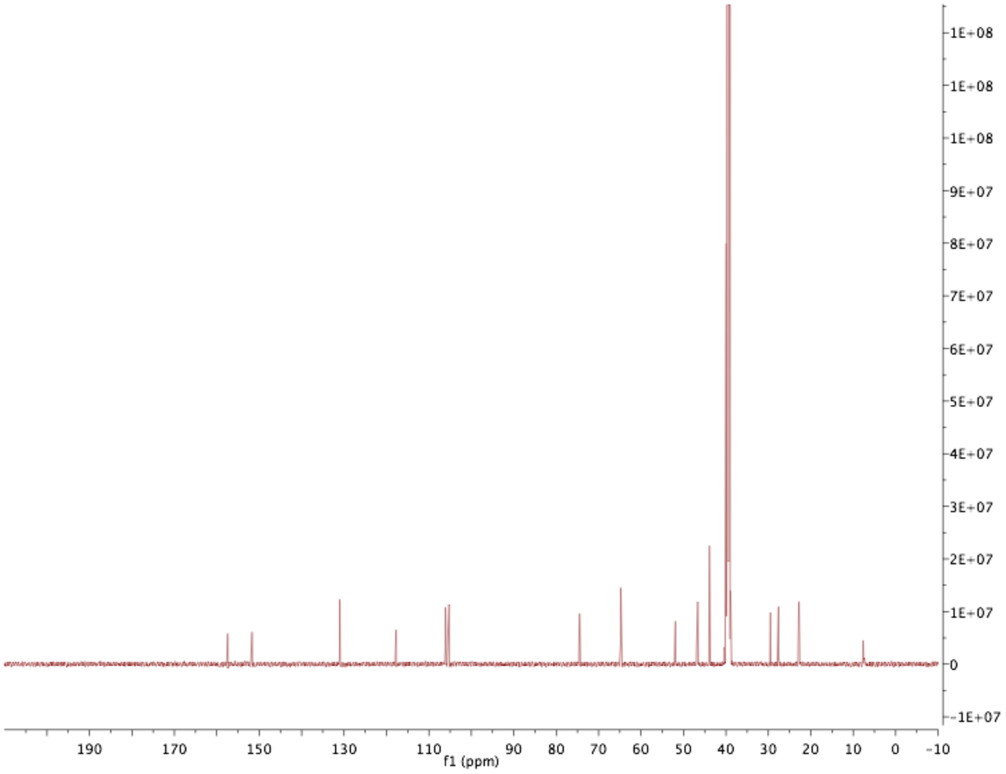

**5{10}**

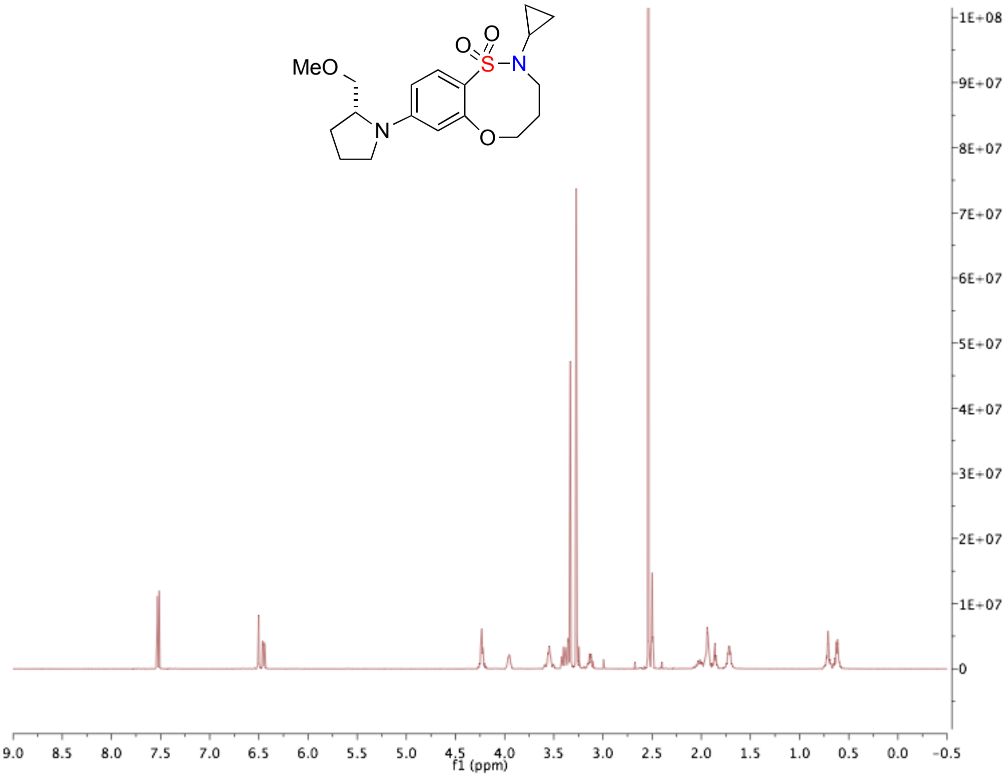
MeO
N
O
S
N
O
1E+08
9E+07
8E+07
7E+07
6E+07
5E+07
4E+07
3E+07
2E+07
1E+07
0
9.0 8.5 8.0 7.5 7.0 6.5 6.0 5.5 5.0 4.5 4.0 3.5 3.0 2.5 2.0 1.5 1.0 0.5 0.0 -0.5
f1 (ppm)

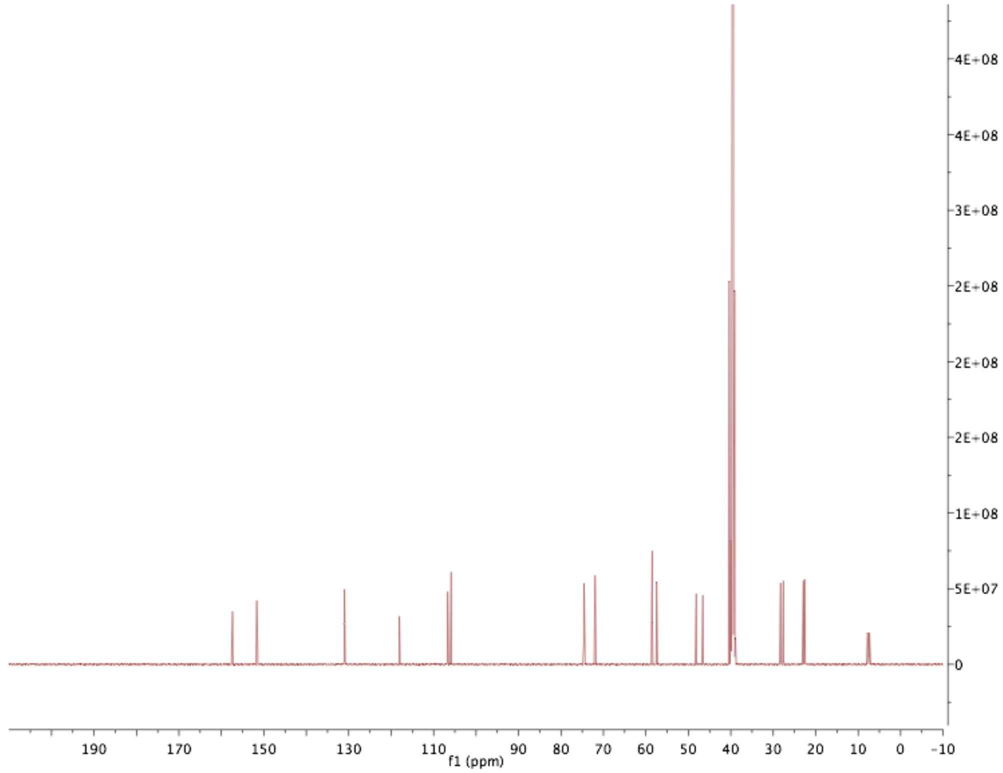
4E+08
4E+08
3E+08
2E+08
2E+08
2E+08
1E+08
5E+07
0
190 170 150 130 110 90 80 70 60 50 40 30 20 10 0 -10
f1 (ppm)

**6{4}**

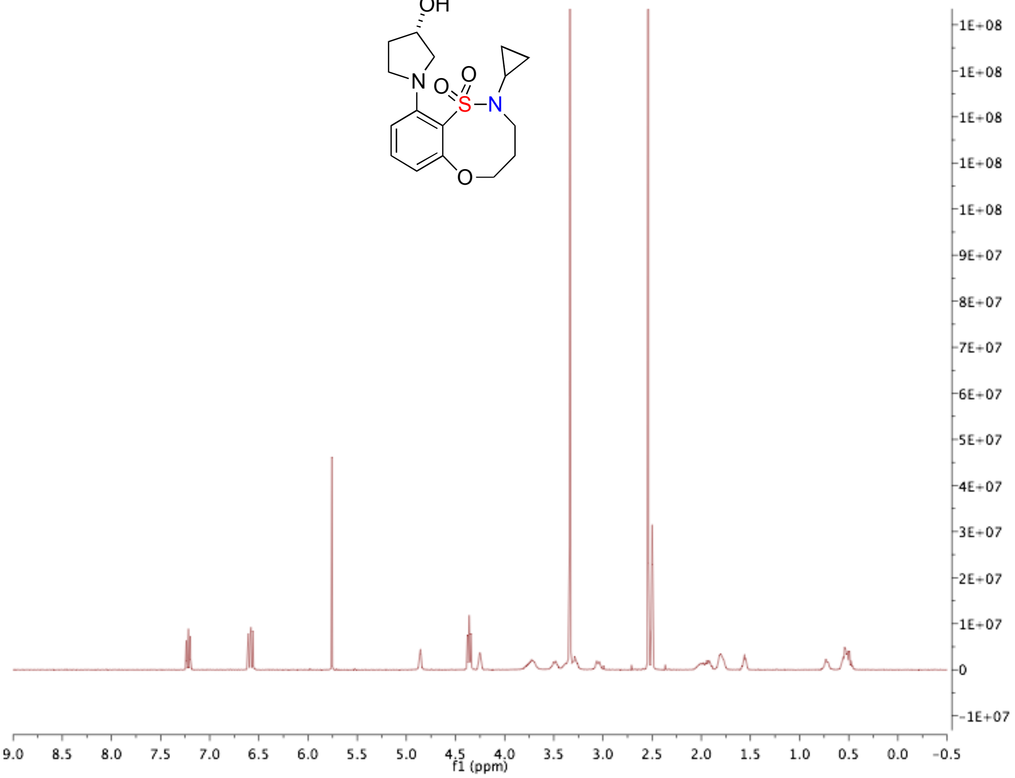
OH
N
O
S
O
N
O
1E+08
9E+07
8E+07
7E+07
6E+07
5E+07
4E+07
3E+07
2E+07
1E+07
0
-1E+07
9.0 8.5 8.0 7.5 7.0 6.5 6.0 5.5 5.0 4.5 4.0 3.5 3.0 2.5 2.0 1.5 1.0 0.5 0.0 -0.5
f1 (ppm)

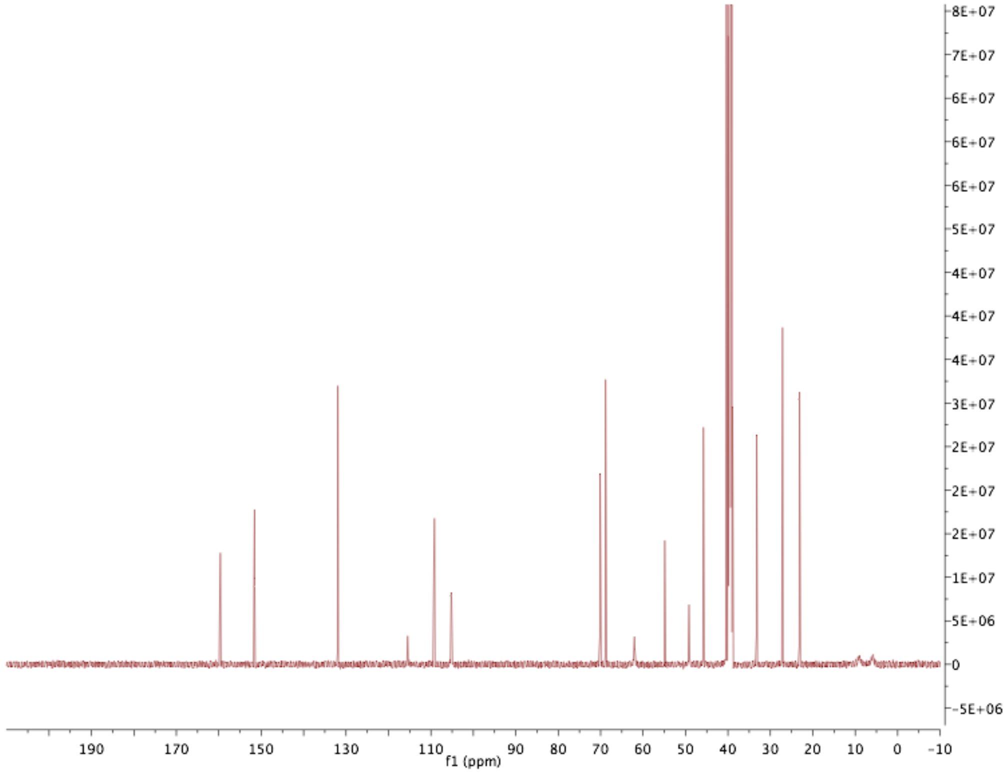
8E+07
7E+07
6E+07
5E+07
4E+07
3E+07
2E+07
1E+07
5E+06
0
-5E+06
190 170 150 130 110 90 80 70 60 50 40 30 20 10 0 -10
f1 (ppm)

**6{6}**

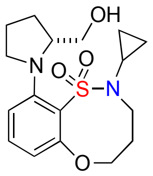


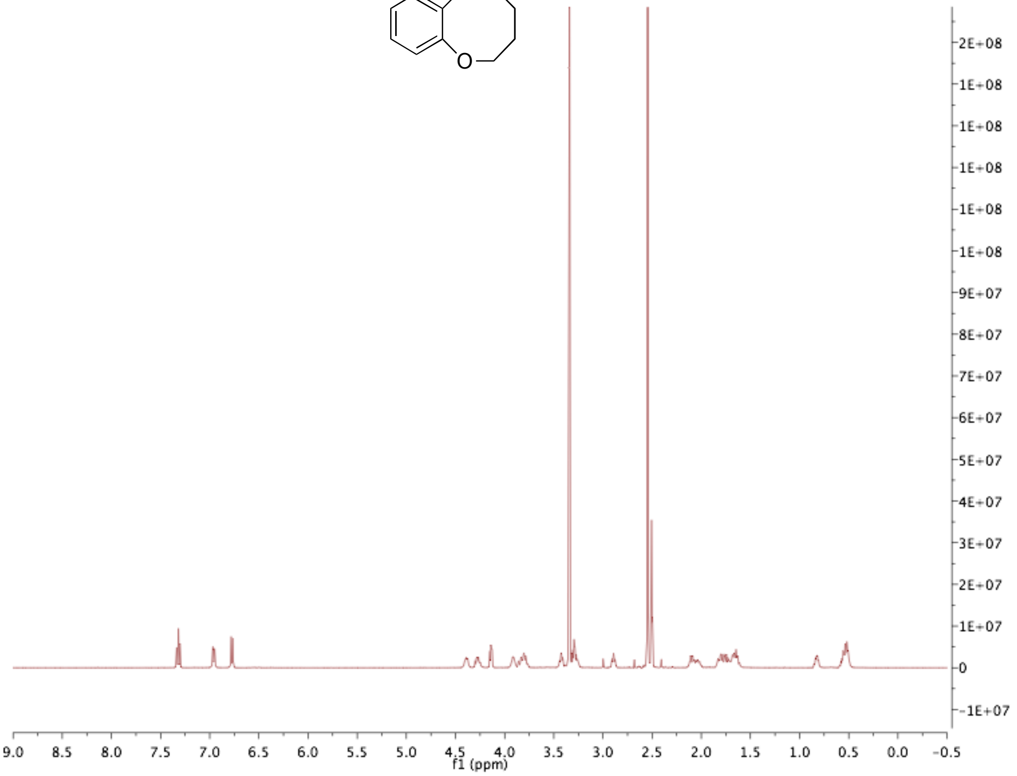


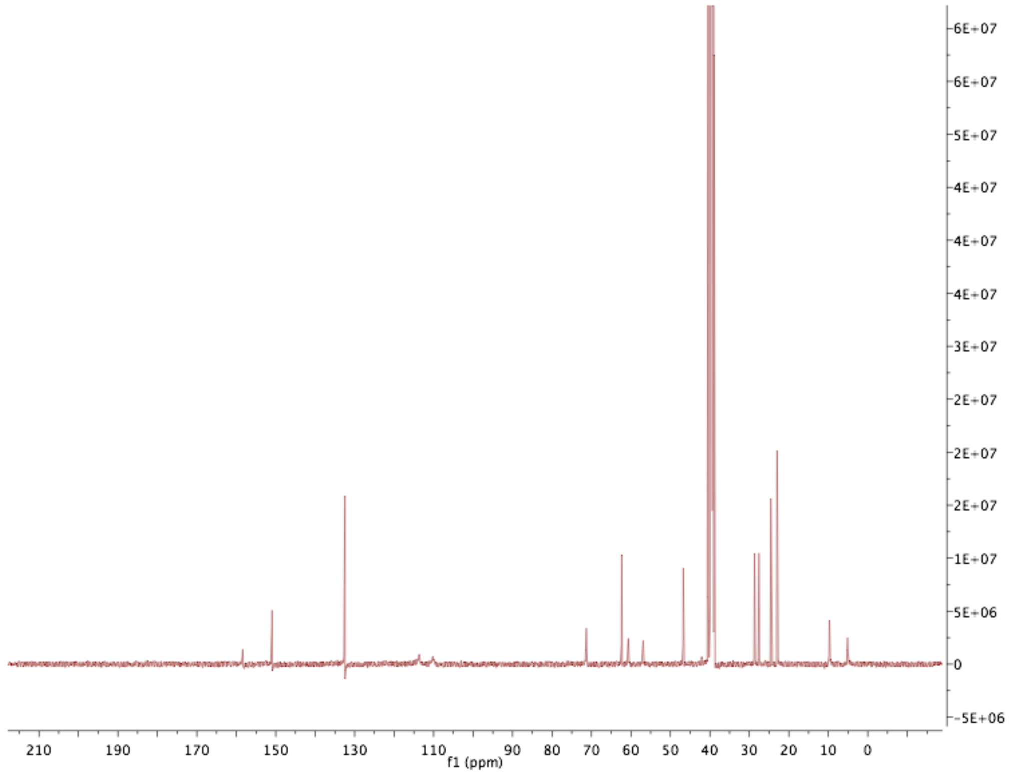

**7{9}**

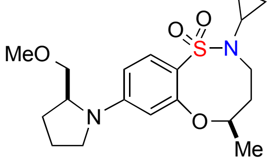
MeO
N
O
O
S
N
O
Me

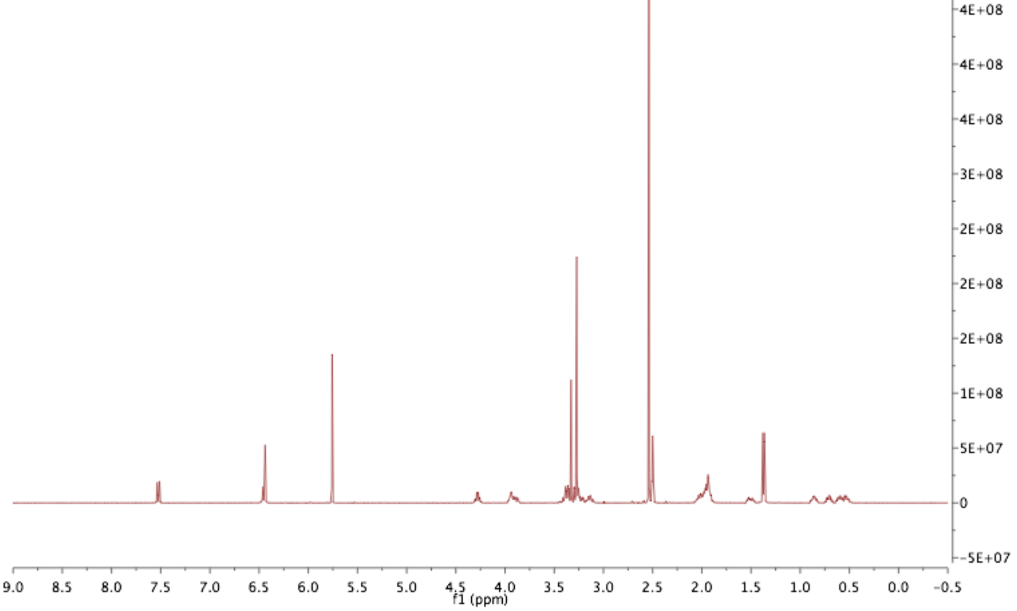
6E+08
6E+08
5E+08
4E+08
4E+08
4E+08
3E+08
2E+08
2E+08
2E+08
1E+08
5E+07
0
-5E+07
9.0 8.5 8.0 7.5 7.0 6.5 6.0 5.5 5.0 4.5 4.0 3.5 3.0 2.5 2.0 1.5 1.0 0.5 0.0 -0.5
f1 (ppm)

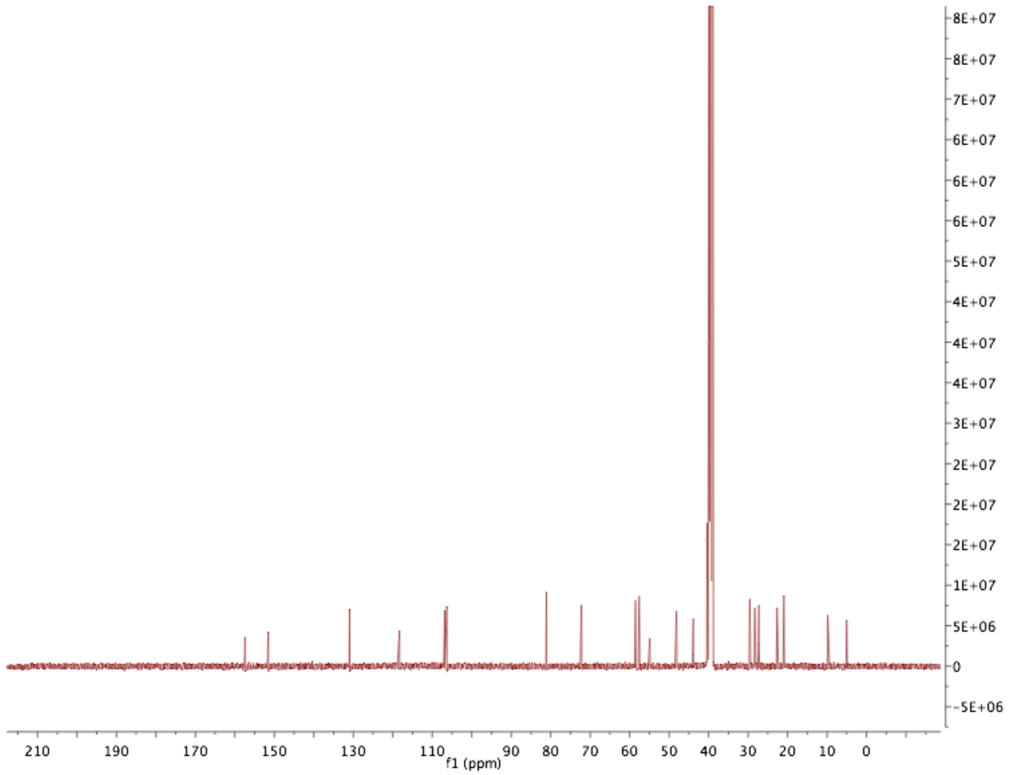
8E+07
8E+07
7E+07
6E+07
6E+07
6E+07
5E+07
4E+07
4E+07
4E+07
3E+07
2E+07
2E+07
2E+07
1E+07
5E+06
0
-5E+06
210 190 170 150 130 110 90 80 70 60 50 40 30 20 10 0
f1 (ppm)

**8{2}**

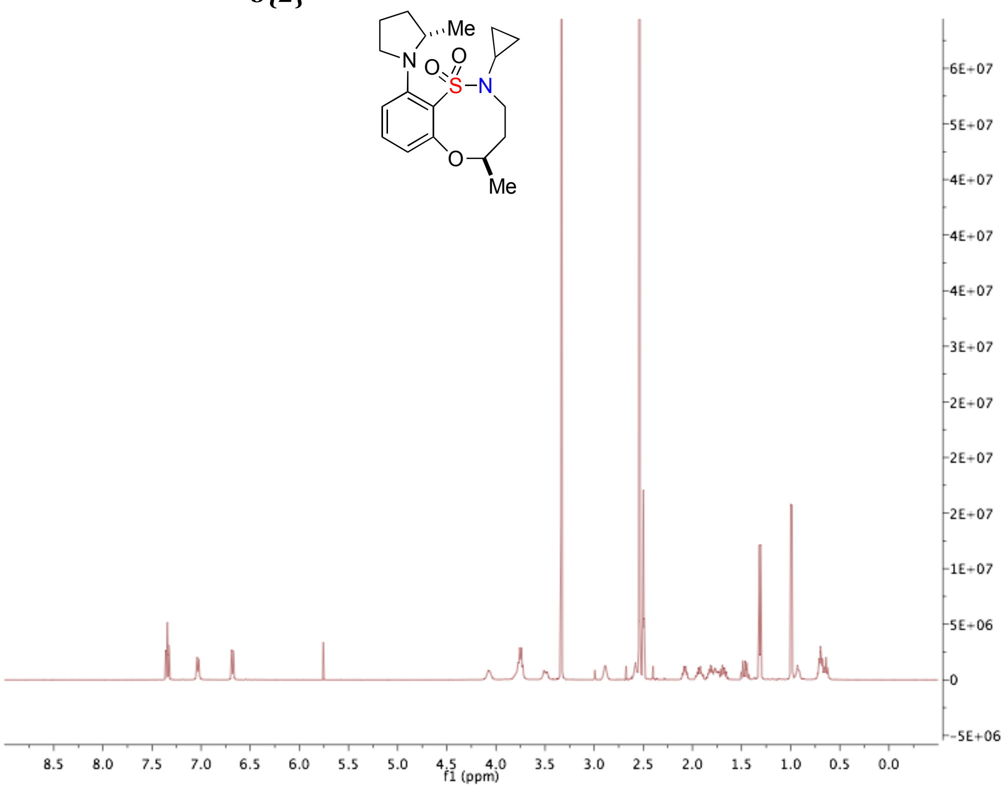


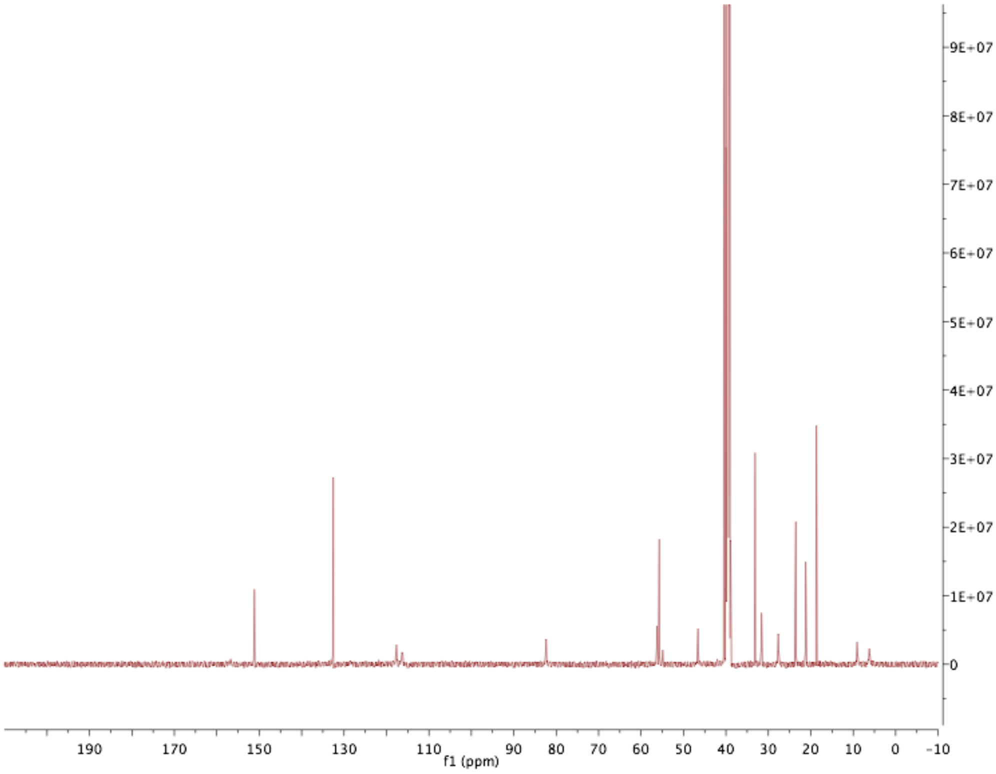

**8{7}**

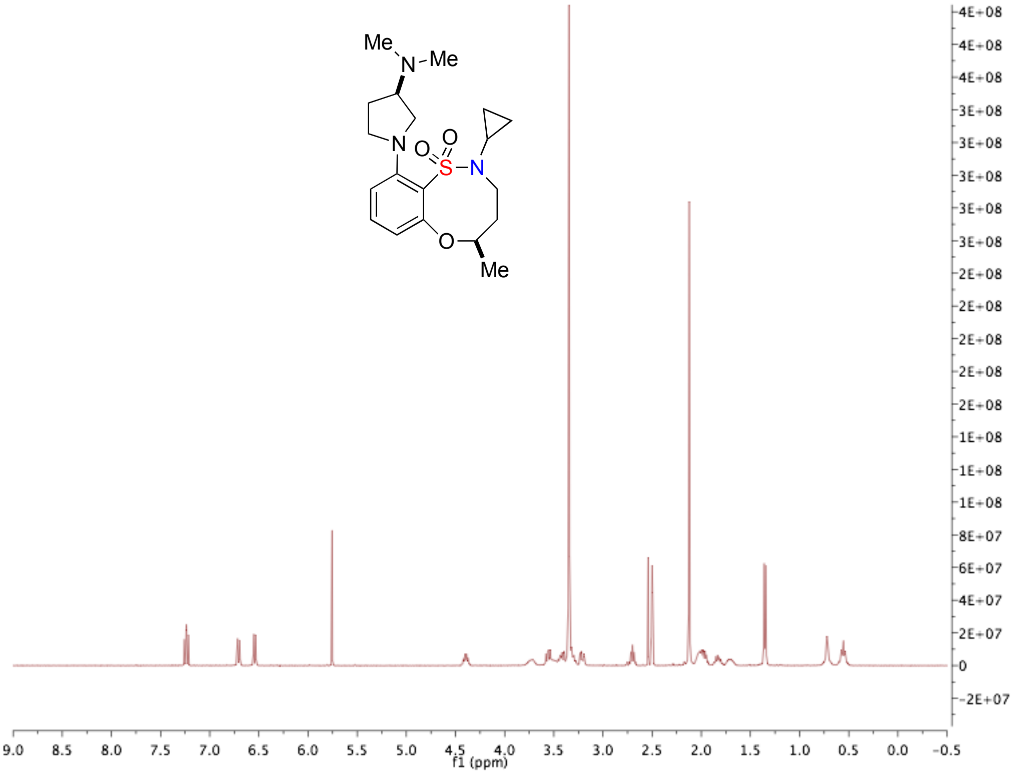


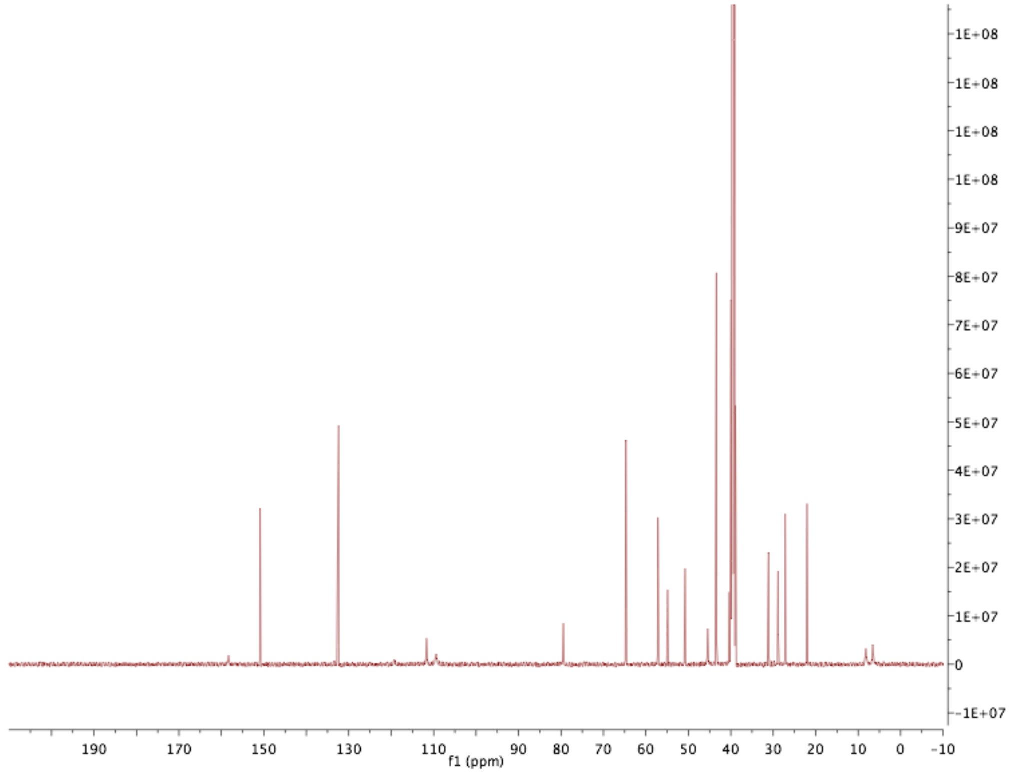

**1{2}**

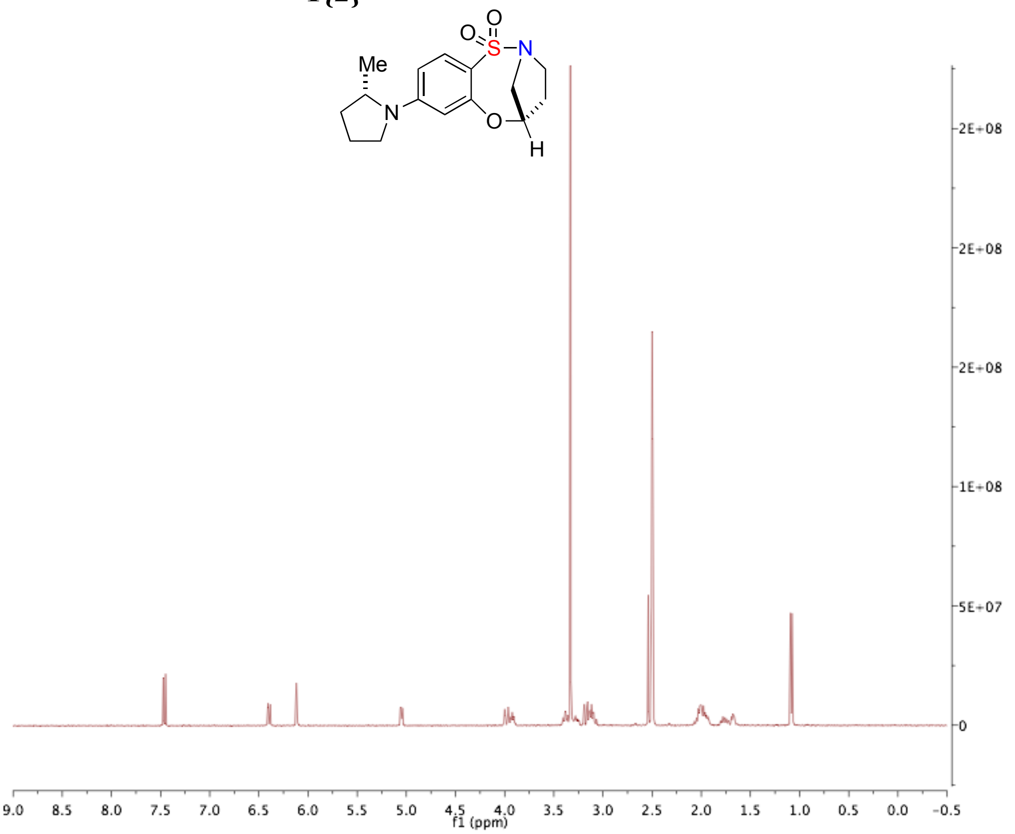
Me
N
O
S
N
O
O
H
2E+08
2E+08
2E+08
1E+08
5E+07
0
9.0 8.5 8.0 7.5 7.0 6.5 6.0 5.5 5.0 4.5 4.0 3.5 3.0 2.5 2.0 1.5 1.0 0.5 0.0 -0.5
f1 (ppm)

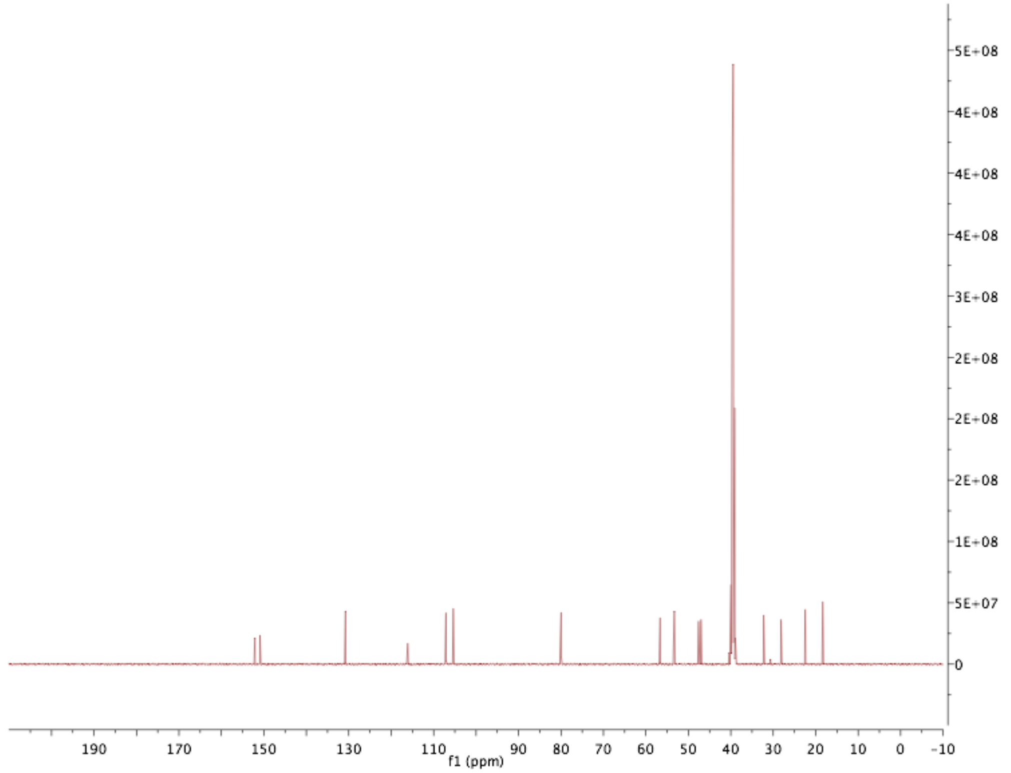
5E+08
4E+08
4E+08
4E+08
3E+08
2E+08
2E+08
2E+08
1E+08
5E+07
0
190 170 150 130 110 90 80 70 60 50 40 30 20 10 0 -10
f1 (ppm)

**1{4}**

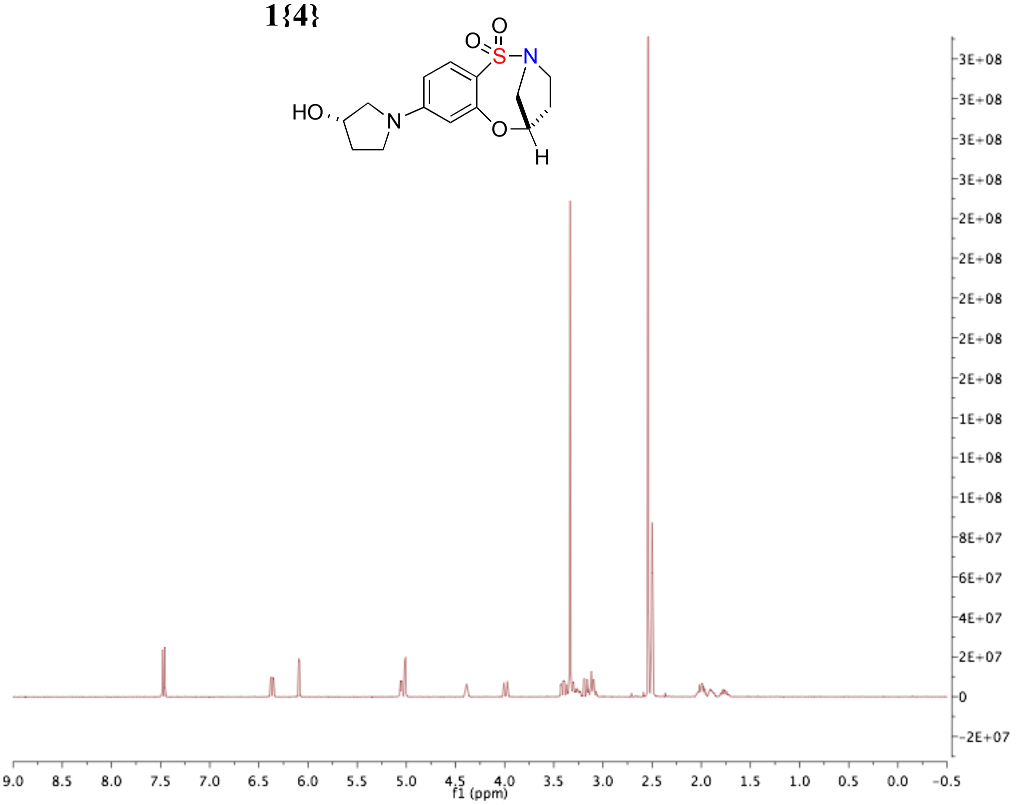

9.0 8.5 8.0 7.5 7.0 6.5 6.0 5.5 5.0 4.5 4.0 3.5 3.0 2.5 2.0 1.5 1.0 0.5 0.0 -0.5
f1 (ppm)


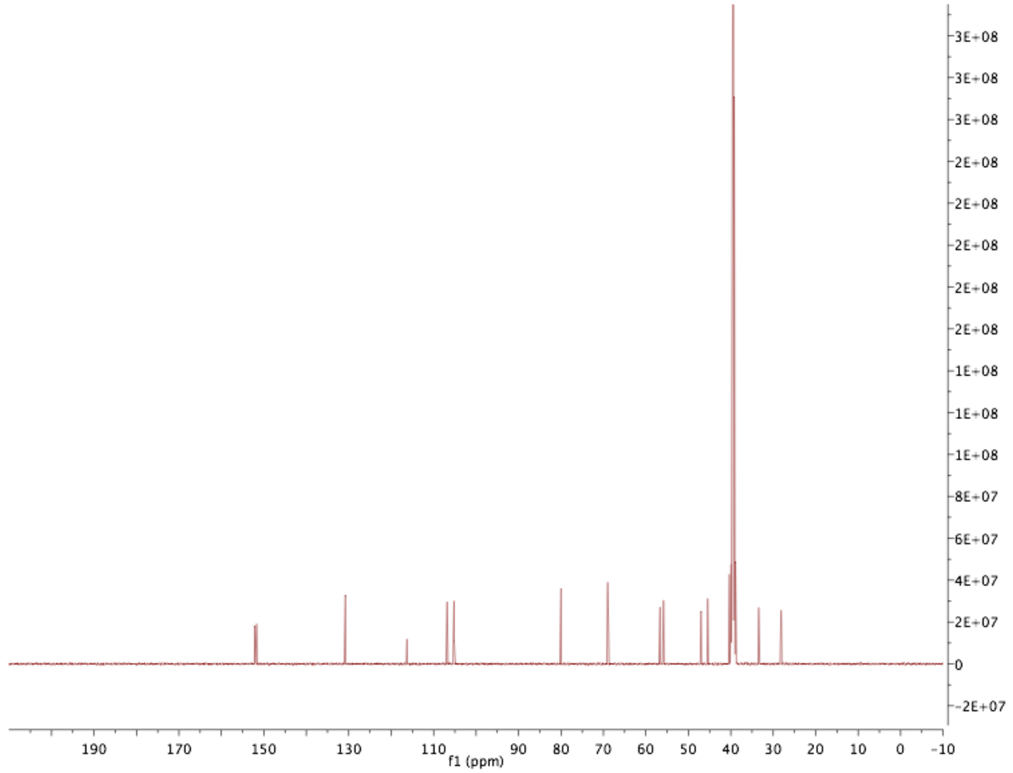

190 170 150 130 110 90 80 70 60 50 40 30 20 10 0 -10
f1 (ppm)

**1{6}**

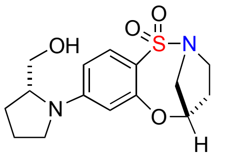

OH
N
O
S
N
O
O
H


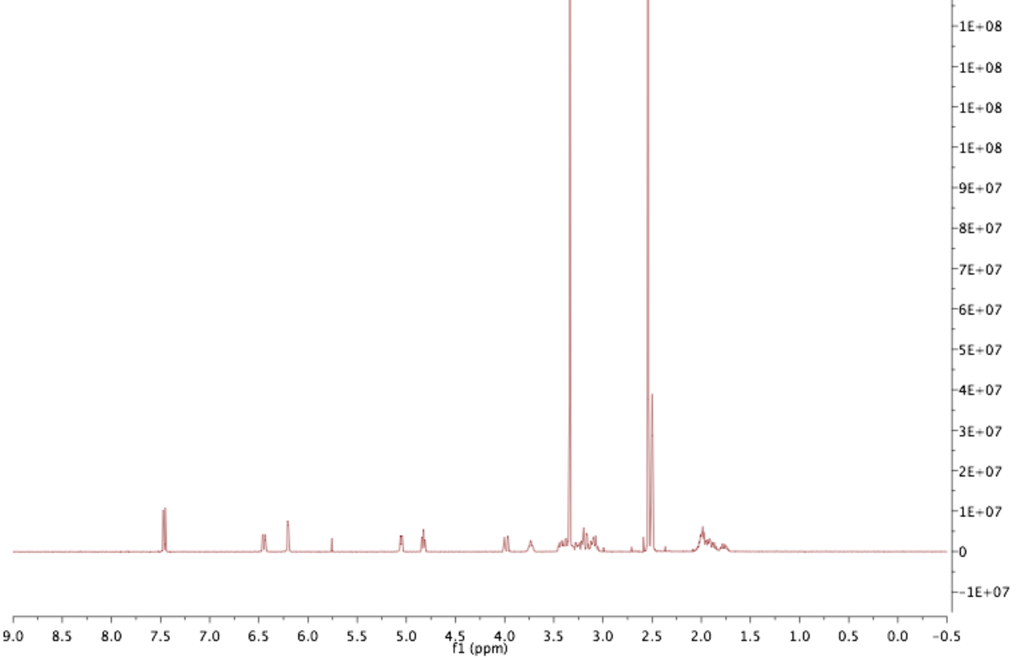

2E+08
2E+08
1E+08
1E+08
1E+08
1E+08
1E+08
9E+07
8E+07
7E+07
6E+07
5E+07
4E+07
3E+07
2E+07
1E+07
0
-1E+07
9.0 8.5 8.0 7.5 7.0 6.5 6.0 5.5 5.0 4.5 4.0 3.5 3.0 2.5 2.0 1.5 1.0 0.5 0.0 -0.5
f1 (ppm)


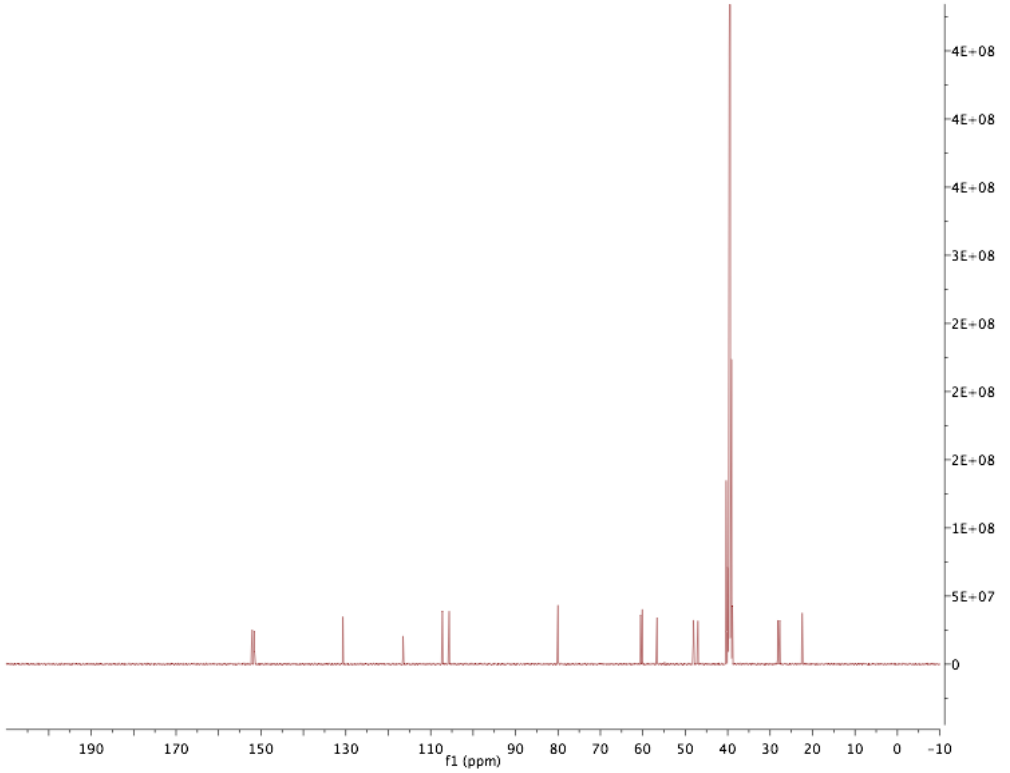

4E+08
4E+08
4E+08
3E+08
2E+08
2E+08
2E+08
1E+08
5E+07
0
190 170 150 130 110 90 80 70 60 50 40 30 20 10 0 -10
f1 (ppm)

**1{9}**

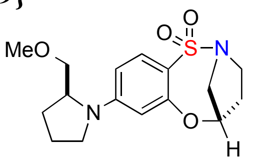
MeO
N
O
S
N
O
H

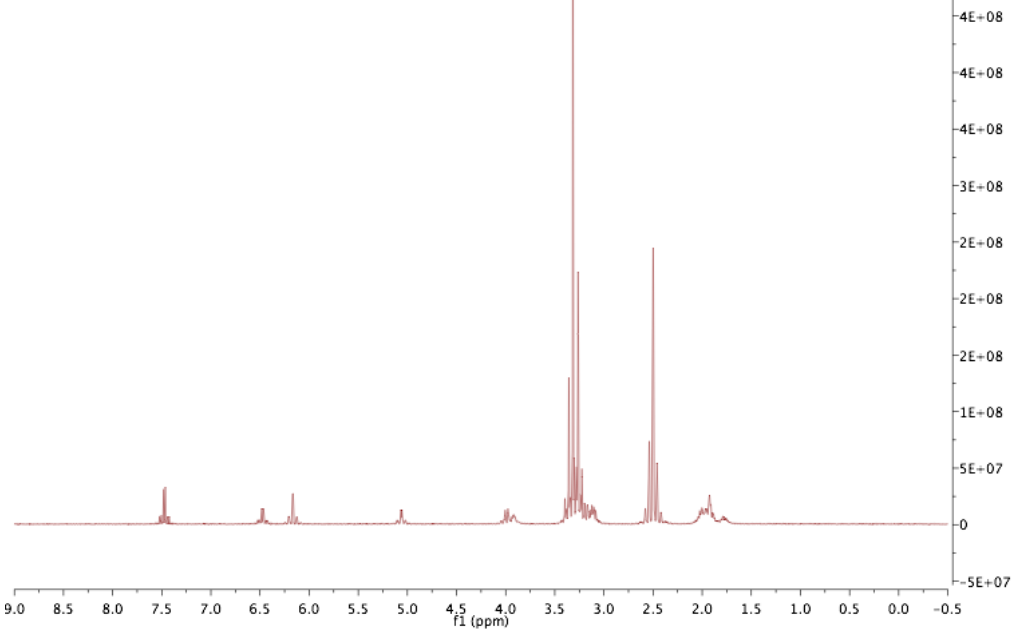
9.0 8.5 8.0 7.5 7.0 6.5 6.0 5.5 5.0 4.5 4.0 3.5 3.0 2.5 2.0 1.5 1.0 0.5 0.0 -0.5
f1 (ppm)
6E+08
5E+08
4E+08
4E+08
4E+08
3E+08
2E+08
2E+08
2E+08
1E+08
5E+07
0
-5E+07

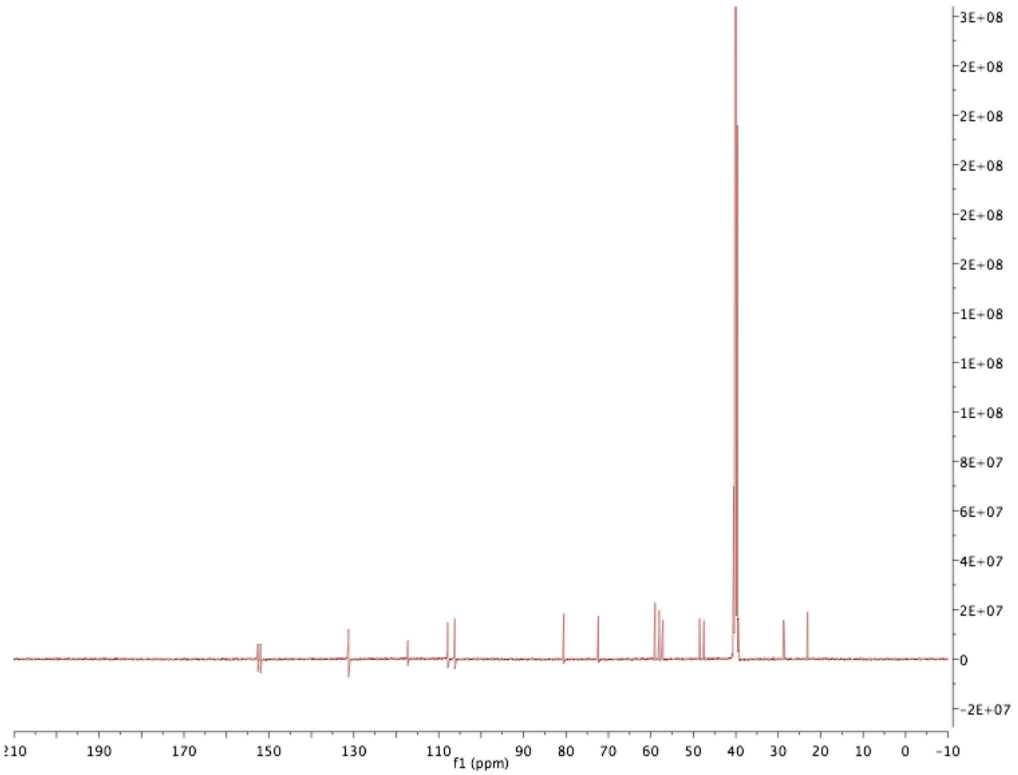
210 190 170 150 130 110 90 80 70 60 50 40 30 20 10 0 -10
f1 (ppm)
3E+08
2E+08
2E+08
2E+08
2E+08
2E+08
1E+08
1E+08
1E+08
8E+07
6E+07
4E+07
2E+07
0
-2E+07

**2{3}**

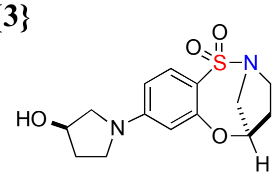


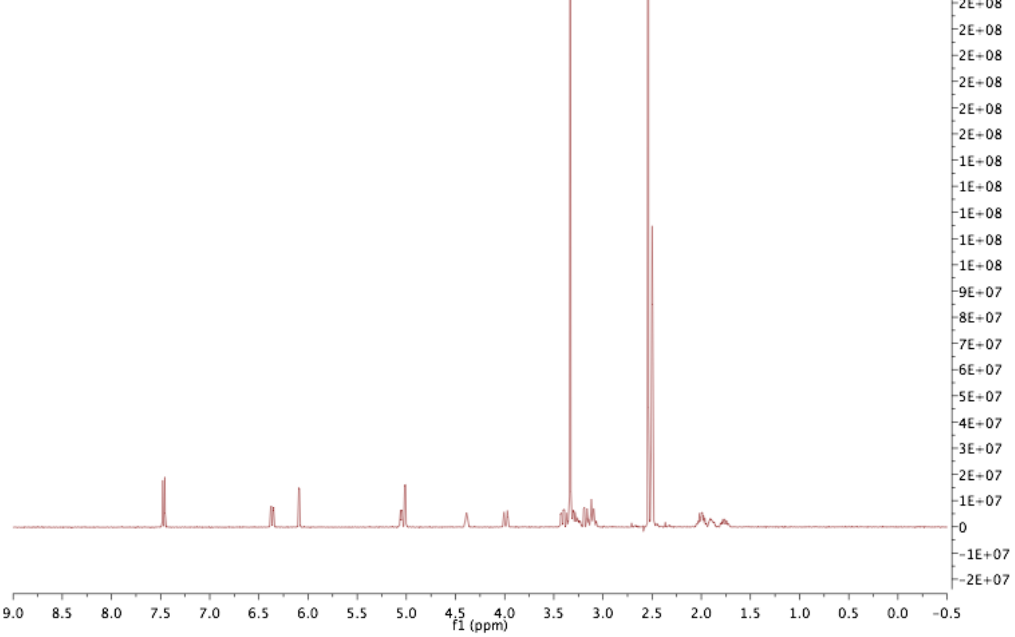


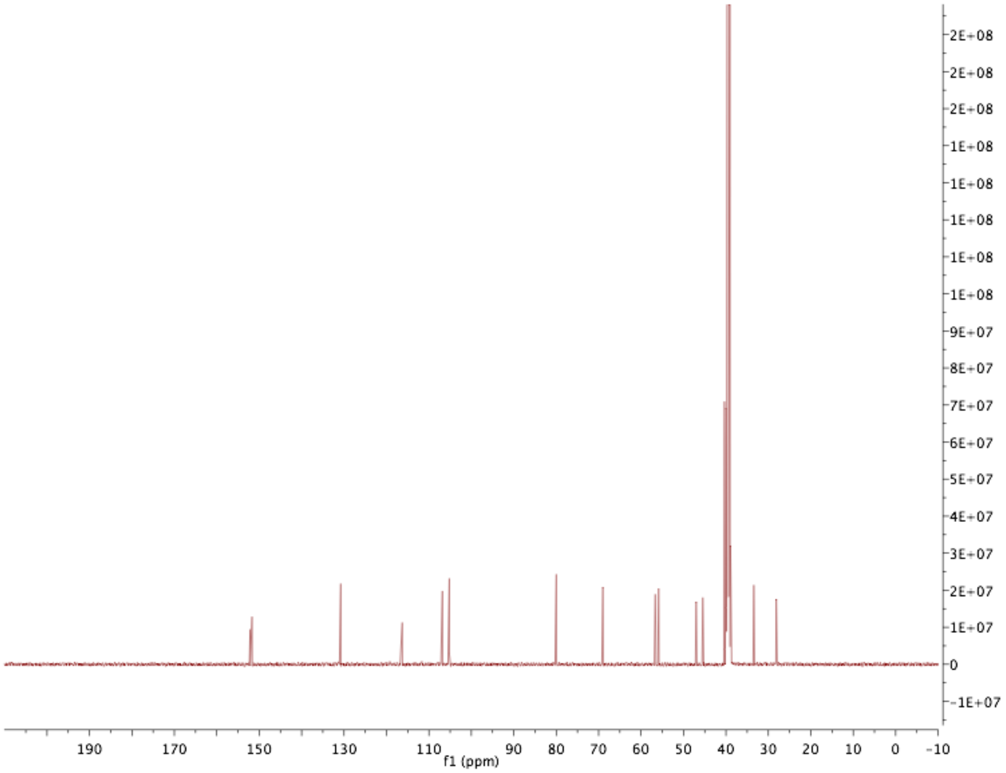

**3{3}**

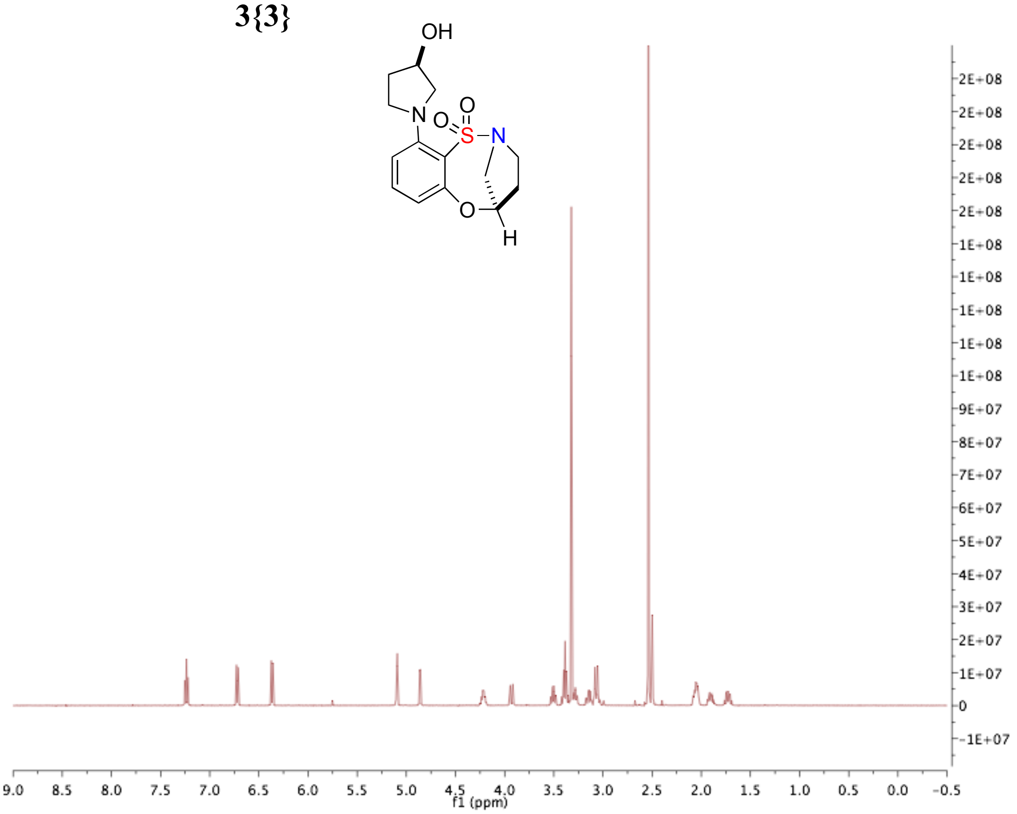


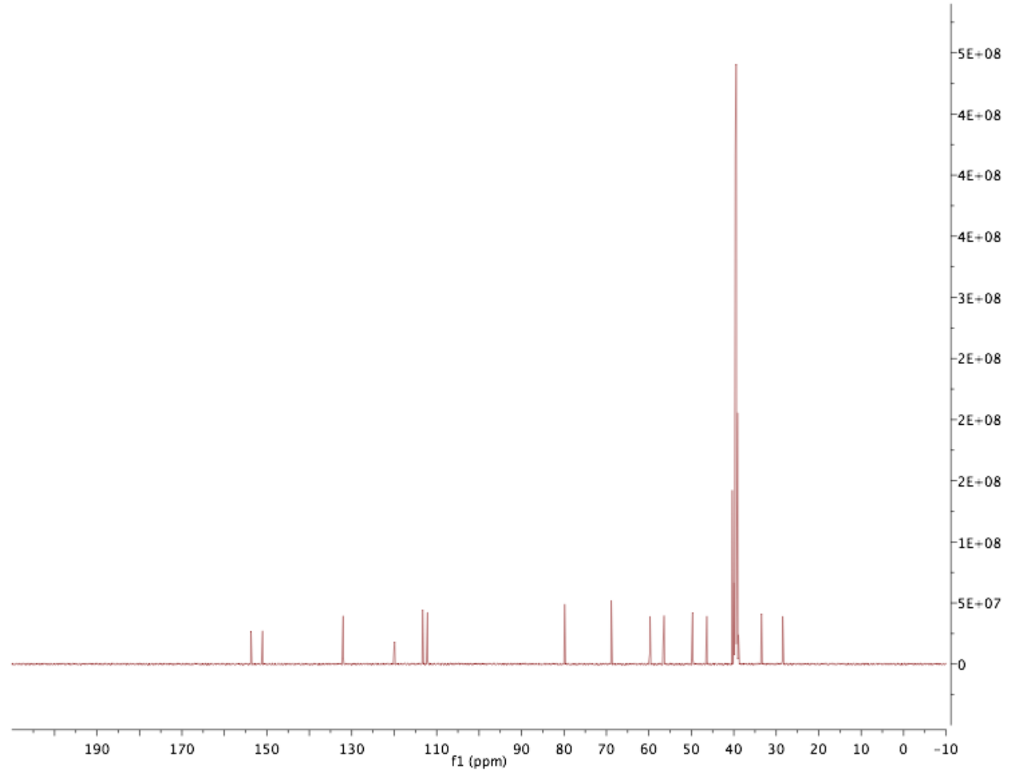

**4{3}**

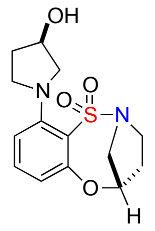


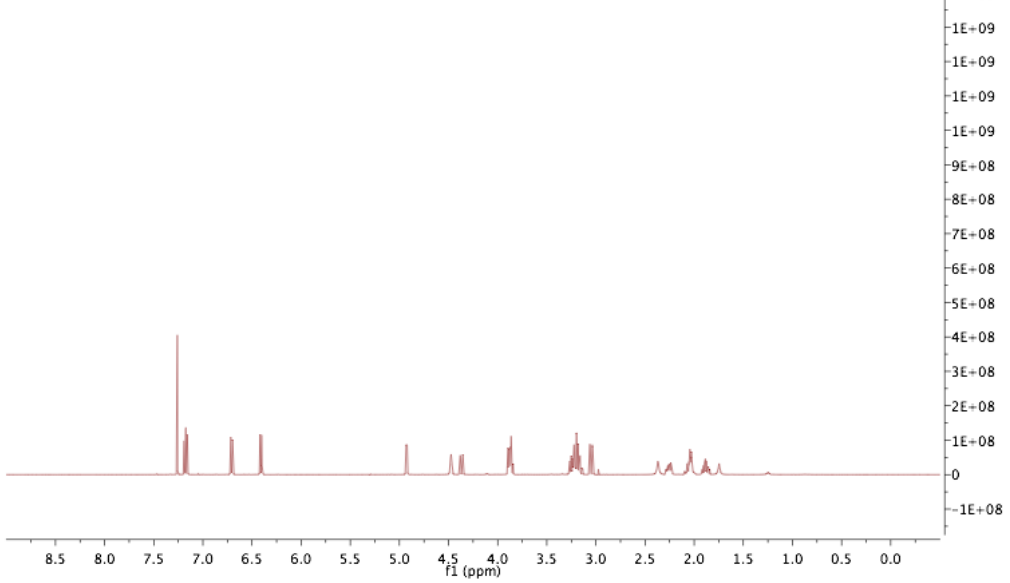


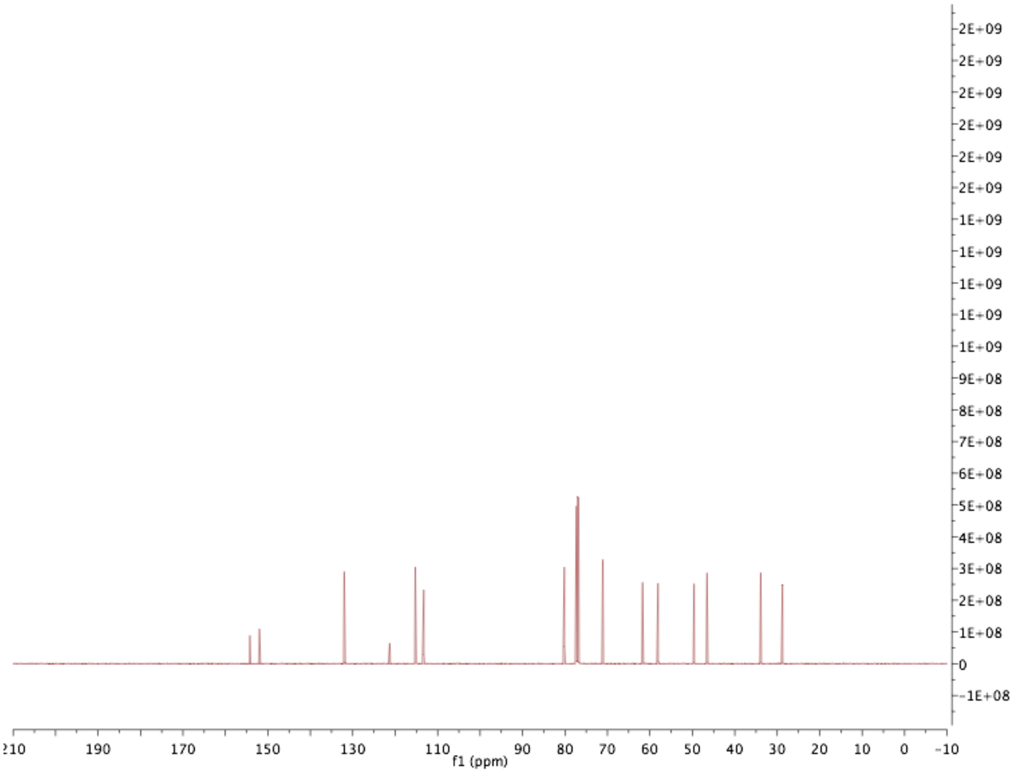

**4{1}**

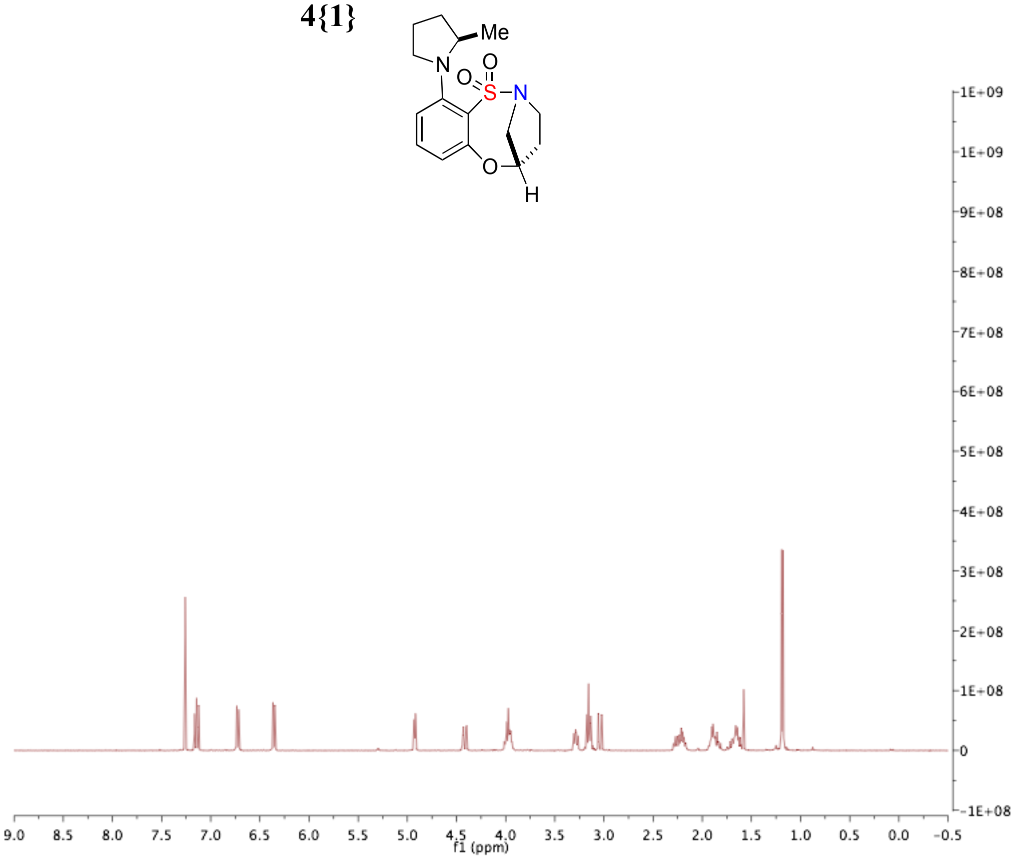


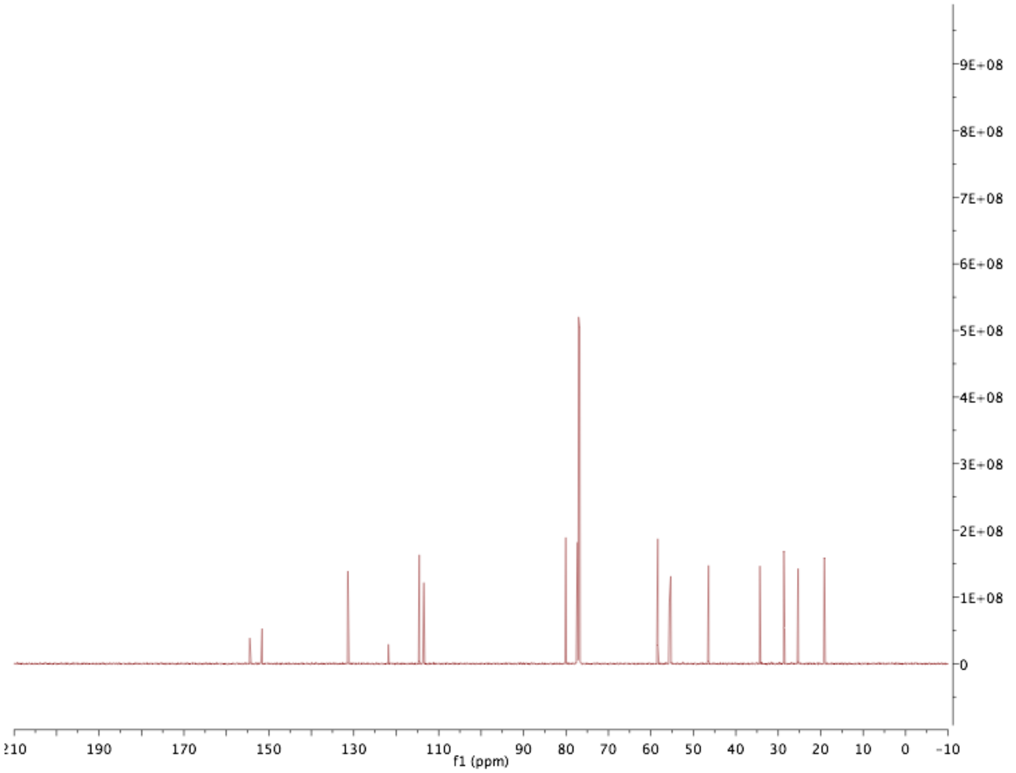

**4{5}**

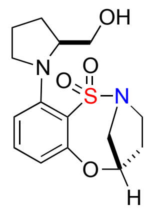

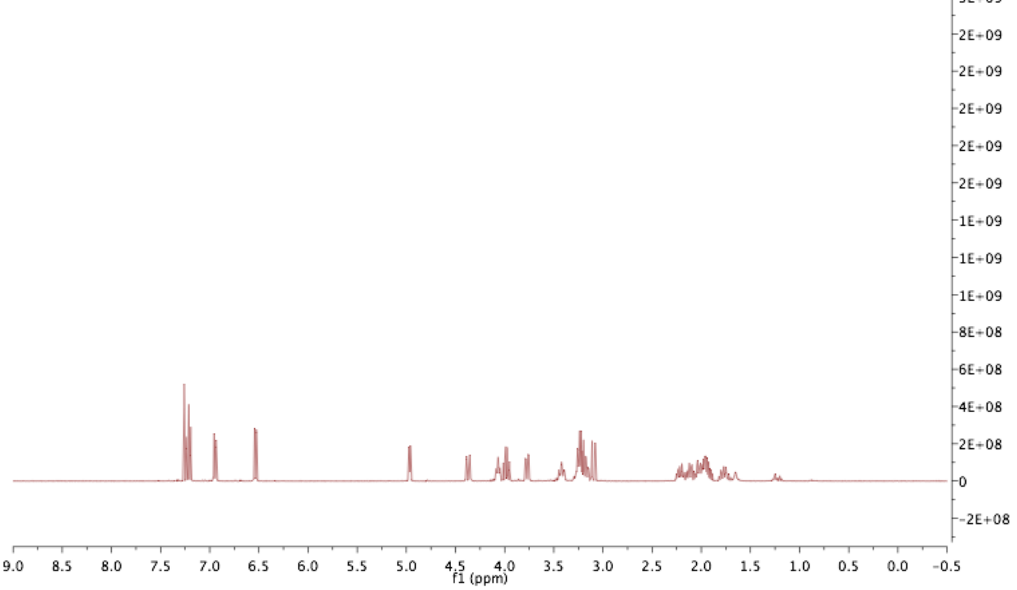


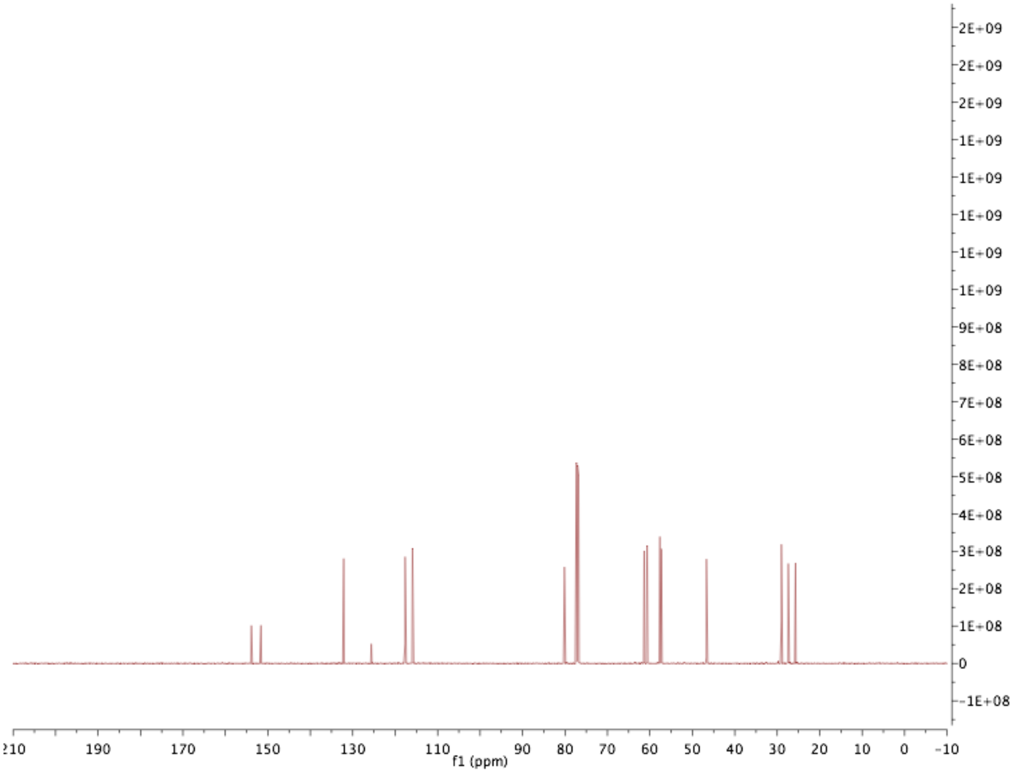

**4{8}**

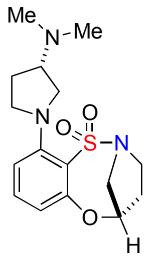


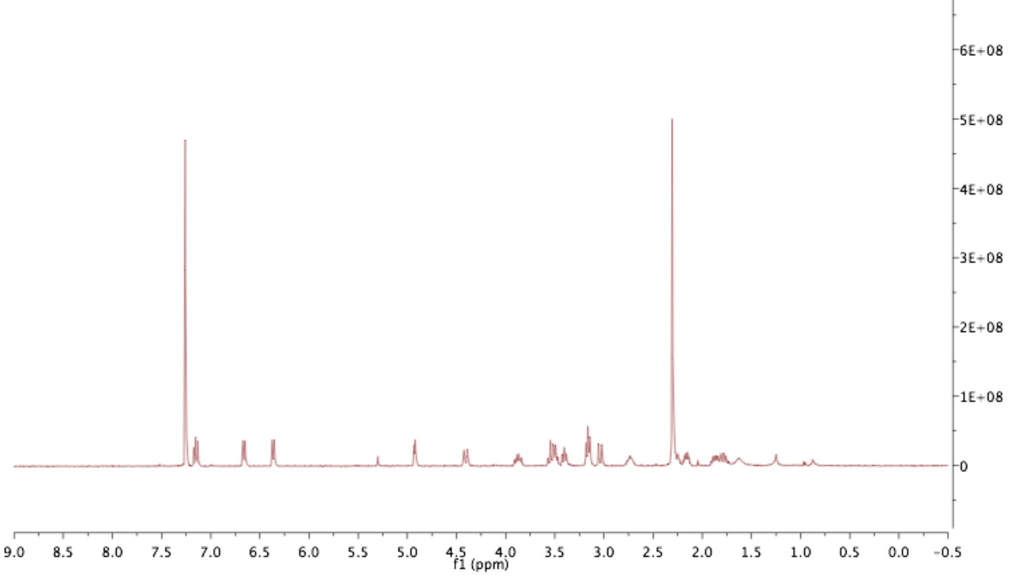


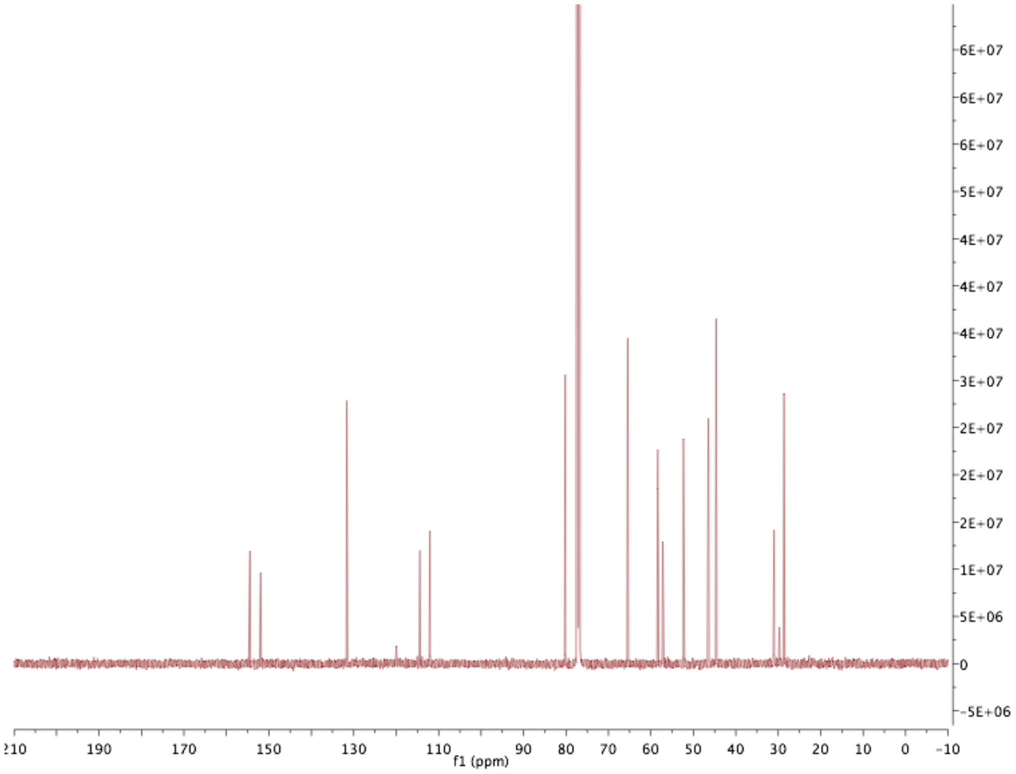

Table of mass spectroscopy data, final mass and purity for all library compounds.

| Comp. | HRMS expected $m/z$ $(M)^+$ | HRMS found $m/z$ $(M + H)^+$ | Mass (mg)[1] | Purity (%) |
|---|---|---|---|---|
| **1{1}** | 308.1195 | 309.1271 | 71.7 | 100 |
| **1{2}** | 308.1195 | 309.1243 | 73.4 | 100 |
| **1{3}** | 310.0987 | 311.1057 | 79.5 | 99.8 |
| **1{4}** | 310.0987 | 311.1032 | 76.7 | 99.8 |
| **1{5}** | 324.1144 | 325.1189 | 73.6 | 99.7 |
| **1{6}** | 324.1144 | 325.1183 | 90.2 | 99.6 |
| **1{7}** | 337.1460 | 338.1500 | 21.2 | 99.1 |
| **1{8}** | 337.1460 | 338.1493 | 94.9 | 100 |
| **1{9}** | 338.1300 | 339.1345 | 84.7 | 100 |
| **1{10}** | 338.1300 | 339.1363 | 93.9 | 100 |
| **2{1}** | 308.1195 | 309.1249 | 80.5 | 99.6 |
| **2{2}** | 308.1195 | 309.1248 | 69.8 | 99.5 |
| **2{3}** | 310.0987 | 311.1014 | 70.0 | 99.4 |
| **2{4}** | 310.0987 | 311.1046 | 46.1 | 98.7 |
| **2{5}** | 324.1144 | 325.1216 | 83.8 | 99.1 |
| **2{6}** | 324.1144 | 325.1199 | 82.9 | 99 |
| **2{7}** | 337.1460 | 338.1511 | 78.5 | 99.2 |
| **2{8}** | 337.1460 | 338.1511 | 18.2 | 98.5 |
| **2{9}** | 338.1300 | 339.1357 | 39.6 | 99.9 |
| **2{10}** | 338.1300 | 339.1364 | 78.8 | 100 |
| **3{1}** | 308.1195 | 309.1248 | 70.1 | 99.5 |
| **3{2}** | 308.1195 | 309.1262 | 64.5 | 99.6 |
| **3{3}** | 310.0987 | 311.1028 | 49.3 | 100 |
| **3{4}** | 310.0987 | 311.1028 | 63.9 | 99.3 |
| **3{5}** | 324.1144 | 325.1205 | 72.1 | 99.6 |
| **3{6}** | 324.1144 | 325.1203 | 65.3 | 100 |
| **3{7}** | 337.1460 | 338.1520 | 69.4 | 99.1 |

| | | | | |
|---|---|---|---|---|
| **3{8}** | 337.1460 | 338.1519 | 69.7 | 98.6 |
| **3{9}** | 338.1300 | 339.1353 | 80.6 | 99.7 |
| **3{10}** | 338.1300 | 339.1356 | 69.4 | 100 |
| **4{1}** | 308.1195 | 309.1259 | 70.8 | 99.1 |
| **4{2}** | 308.1195 | 309.1260 | 70.4 | 99 |
| **4{3}** | 310.0987 | 311.1051 | 54.1 | 99.7 |
| **4{4}** | 310.0987 | 311.1035 | 41.3 | 99.5 |
| **4{5}** | 324.1144 | 325.1194 | 72.9 | 100 |
| **4{6}** | 324.1144 | 325.1208 | 82.2 | 99.5 |
| **4{7}** | 337.1460 | 338.1516 | 83.7 | 98.6 |
| **4{8}** | 337.1460 | 338.1517 | 86.3 | 99.1 |
| **4{9}** | 338.1300 | 339.1367 | 88.5 | 99.1 |
| **4{10}** | 338.1300 | 339.1368 | 83.5 | 99.7 |
| **5{1}** | 336.1508 | 337.1580 | 79.8 | 100 |
| **5{2}** | 336.1508 | 337.1584 | 79.4 | 100 |
| **5{3}** | 338.1300 | 339.1339 | 75.8 | 100 |
| **5{4}** | 338.1300 | 339.1344 | 79.1 | 100 |
| **5{5}** | 352.1457 | 353.1510 | 85.7 | 100 |
| **5{6}** | 352.1457 | 353.1508 | 83.1 | 100 |
| **5{7}** | 365.1773 | 366.1813 | 18.7 | 98.2 |
| **5{8}** | 365.1773 | 366.1826 | 49.2 | 99.9 |
| **5{9}** | 366.1613 | 367.1661 | 85.3 | 99.1 |
| **5{10}** | 366.1613 | 367.1680 | 83.7 | 99.1 |
| **6{1}** | 336.1508 | 337.1581 | 66.1 | 98.8 |
| **6{2}** | 336.1508 | 337.1578 | 69.5 | 98 |
| **6{3}** | 338.1300 | 339.1376 | 77.2 | 97.6 |
| **6{4}** | 338.1300 | 339.1356 | 61.0 | 97.7 |
| **6{5}** | 352.1457 | 353.1501 | 68.6 | 99.7 |
| **6{6}** | 352.1457 | 353.1519 | 67.7 | 99.6 |
| **6{7}** | 365.1773 | 366.1834 | 76.2 | 96.9 |
| **6{8}** | 365.1773 | 366.1840 | 75.2 | 98.1 |

| | | | | |
|---|---|---|---|---|
| **6{9}** | 366.1613 | 367.1668 | 76.1 | 100 |
| **6{10}** | 366.1613 | 367.1677 | 81.5 | 99.9 |
| **7{1}** | 350.1664 | 351.1730 | 72.7 | 99 |
| **7{2}** | 350.1664 | 351.1709 | 44.2 | 99.5 |
| **7{3}** | 352.1457 | 353.1500 | 74.0 | 97.7 |
| **7{4}** | 352.1457 | 353.1539 | 61.8 | 97.7 |
| **7{5}** | 366.1613 | 367.1680 | 76.9 | 98.7 |
| **7{6}** | 366.1613 | 367.1686 | 76.7 | 98.6 |
| **7{7}** | 379.1930 | 380.1978 | 46.5 | 99.5 |
| **7{8}** | 379.1930 | 380.1995 | 77.9 | 99.3 |
| **7{9}** | 380.1770 | 381.1831 | 67.4 | 99.9 |
| **7{10}** | 380.1770 | 381.1830 | 92.6 | 100 |
| **8{1}** | 350.1664 | 351.1712 | 50.8 | 98.6 |
| **8{2}** | 350.1664 | 351.1700 | 33.9 | 98.6 |
| **8{3}** | 352.1457 | 353.1500 | 49.9 | 99.6 |
| **8{4}** | 352.1457 | 353.1517 | 42.7 | 99.6 |
| **8{5}** | 366.1613 | 367.1684 | 70.1 | 99.6 |
| **8{6}** | 366.1613 | 367.1682 | 46.1 | 99.4 |
| **8{7}** | 379.1930 | 380.1976 | 37.9 | 95.5 |
| **8{8}** | 379.1930 | 380.1981 | 127.4 | 97.3 |
| **8{9}** | 380.1770 | 381.1844 | 34.6 | 99.6 |
| **8{10}** | 380.1770 | 381.1821 | 22.3 | 98.9 |

[1]Low yields obtained are due to several reasons including:

1. Peaks having bad shouldering problems
2. Only a small amount of the peak that satisfies the 90% purity threshold
3. Mechanical/instrumental error: over-pressured.

## In silico analysis

Sketched electronic versions of the library compounds were imported into the Tripos Molecular Spreadsheet [2] wherein standard Lipinski's rule of five parameters (molecular weight, ClogP, number of H-acceptors, and number of H-donors [3]) plus the number of rotatable bonds and polar surface area were computed. Lipinski violations were specified according to molecular weight > 500, ClogP > 5.0, number of acceptors > 10, number of donors > 5, and number of rotatable bonds > 5. The structures were then exported into SDF format and converted into three-dimensional protonated structures via Concord [4]. Absorption, distribution, metabolism and excretion (ADME) profiles of these compounds were then generated via Volsurf [5]. Descriptors were generated by using three probes (water, hydrophobic and carbonyl oxygen) with a grid space distribution of 1.0 Å. Predictions were then projected onto internal ADME models at the five-component level. Finally, diversity analysis was carried out by using DiverseSolutions [6] using standard H-aware 3D BCUT descriptors. The library was then projected onto a chemical space defined by the following descriptors: gastchrg_invdist2_000.550_K_L, gastchrg_invdist6_000.500_K_H, haccept_invdist2_001.000_K_H, tabpolar_invdist_000.250_K_H, tabpolar_invdist_000.500_K_L and populated (for comparison) by a recent version of the MLSMR screening set (ca. 7/2010; ~330,000 unique chemical structures). Diversity scores (*div(A)*) for our library were then generated for each of our compounds (*A*) according to the expression:

$$div(A) = \frac{pop[Cell(A)]}{\sum_{i \in Occ} pop(i) / N_{occ}}$$

where $N_{occ}$ is the number of cells occupied by PubChem compounds in an evenly distributed 10×10×10×10×10 grid decomposition of the chemistry space, and *pop(i)* is the population of cell *i*.

| Molecule | CLOGP | Mol.Wt | Acceptor | Donor | Rot Bond | LIP_VIOLS | PSA | DIVS | BBB | SOLY | CACO2 | SP_S | SP_P | PB | VOLD | HERG | Sol_DMSO | METSTAB |
|---|---|---|---|---|---|---|---|---|---|---|---|---|---|---|---|---|---|---|
| 1{1} | 2.31 | 308.40 | 4 | 1 | 1 | 0 | 82.29 | 0.12 | 0.81 | -4.32 | 0.86 | 0.28 | 0.64 | 76.01 | -0.27 | 0.52 | 0.69 | -0.18 |
| 1{2} | 2.31 | 308.40 | 4 | 1 | 1 | 0 | 82.30 | 0.12 | 0.79 | -4.24 | 0.78 | 0.26 | 0.66 | 76.31 | -0.23 | 0.46 | 0.77 | -0.22 |
| 1{3} | 0.45 | 310.37 | 5 | 2 | 2 | 0 | 131.01 | 0.13 | -0.19 | -3.64 | 0.29 | 0.48 | 0.11 | 64.50 | -0.18 | 0.65 | 1.76 | 0.44 |
| 1{4} | 0.45 | 310.37 | 5 | 2 | 2 | 0 | 130.95 | 0.13 | -0.30 | -3.63 | 0.23 | 0.50 | 0.17 | 63.82 | -0.14 | 0.73 | 1.87 | 0.43 |
| 1{5} | 1.07 | 324.40 | 5 | 2 | 3 | 0 | 134.37 | 0.39 | -0.17 | -3.38 | 0.35 | 0.43 | 0.24 | 62.71 | -0.28 | 0.74 | 1.91 | 0.49 |
| 1{6} | 1.07 | 324.40 | 5 | 2 | 3 | 0 | 130.54 | 0.39 | 0.03 | -3.93 | 0.53 | 0.44 | 0.12 | 64.56 | -0.40 | 0.44 | 1.71 | 0.37 |
| 1{7} | 1.40 | 337.44 | 4 | 2 | 2 | 0 | 85.01 | 0.33 | 0.59 | -4.40 | 0.67 | 0.19 | 0.42 | 74.82 | -0.21 | 0.36 | 0.99 | -0.29 |
| 1{8} | 1.40 | 337.44 | 4 | 2 | 2 | 0 | 85.03 | 0.33 | 0.37 | -4.13 | 0.66 | 0.19 | 0.41 | 74.36 | -0.36 | 0.39 | 0.87 | -0.21 |
| 1{9} | 1.83 | 338.42 | 5 | 1 | 3 | 0 | 91.49 | 0.13 | 0.40 | -3.90 | 0.63 | 0.26 | 0.52 | 69.78 | -0.41 | 0.69 | 1.18 | 0.09 |
| 1{10} | 1.83 | 338.42 | 5 | 1 | 3 | 0 | 90.32 | 0.13 | 0.55 | -4.30 | 0.72 | 0.27 | 0.45 | 74.93 | -0.33 | 0.46 | 1.10 | -0.23 |
| 2{1} | 2.31 | 308.40 | 4 | 1 | 1 | 0 | 82.29 | 0.12 | 0.81 | -4.32 | 0.86 | 0.28 | 0.64 | 76.01 | -0.27 | 0.52 | 0.69 | -0.18 |
| 2{2} | 2.31 | 308.40 | 4 | 1 | 1 | 0 | 82.30 | 0.12 | 0.79 | -4.24 | 0.78 | 0.26 | 0.66 | 76.31 | -0.23 | 0.46 | 0.77 | -0.22 |
| 2{3} | 0.45 | 310.37 | 5 | 2 | 2 | 0 | 131.01 | 0.13 | -0.19 | -3.64 | 0.29 | 0.48 | 0.11 | 64.50 | -0.18 | 0.65 | 1.76 | 0.44 |
| 2{4} | 0.45 | 310.37 | 5 | 2 | 2 | 0 | 130.95 | 0.13 | -0.30 | -3.63 | 0.23 | 0.50 | 0.17 | 63.82 | -0.14 | 0.73 | 1.87 | 0.43 |
| 2{5} | 1.07 | 324.40 | 5 | 2 | 3 | 0 | 134.37 | 0.39 | -0.17 | -3.38 | 0.35 | 0.43 | 0.24 | 62.71 | -0.28 | 0.74 | 1.91 | 0.49 |
| 2{6} | 1.07 | 324.40 | 5 | 2 | 3 | 0 | 130.54 | 0.39 | 0.03 | -3.93 | 0.53 | 0.44 | 0.12 | 64.56 | -0.40 | 0.44 | 1.71 | 0.37 |
| 2{7} | 1.40 | 337.44 | 4 | 2 | 2 | 0 | 85.01 | 0.33 | 0.59 | -4.40 | 0.67 | 0.19 | 0.42 | 74.82 | -0.21 | 0.36 | 0.99 | -0.29 |
| 2{8} | 1.40 | 337.44 | 4 | 2 | 2 | 0 | 85.03 | 0.33 | 0.37 | -4.13 | 0.66 | 0.19 | 0.41 | 74.36 | -0.36 | 0.39 | 0.87 | -0.21 |
| 2{9} | 1.83 | 338.42 | 5 | 1 | 3 | 0 | 91.49 | 0.13 | 0.40 | -3.90 | 0.63 | 0.26 | 0.52 | 69.78 | -0.41 | 0.69 | 1.18 | 0.09 |
| 2{10} | 1.83 | 338.42 | 5 | 1 | 3 | 0 | 90.32 | 0.13 | 0.55 | -4.30 | 0.72 | 0.27 | 0.45 | 74.93 | -0.33 | 0.46 | 1.10 | -0.23 |
| 3{1} | 2.31 | 308.40 | 4 | 1 | 1 | 0 | 49.01 | 0.12 | 1.24 | -4.39 | 1.10 | 0.25 | 0.86 | 86.48 | -0.54 | 0.83 | 0.44 | -0.31 |
| 3{2} | 2.31 | 308.40 | 4 | 1 | 1 | 0 | 61.58 | 0.12 | 0.75 | -4.01 | 1.09 | 0.19 | 0.92 | 81.13 | -0.67 | 0.66 | 0.65 | -0.03 |
| 3{3} | 0.45 | 310.37 | 5 | 2 | 2 | 0 | 113.67 | 0.04 | 0.13 | -3.50 | 0.59 | 0.35 | 0.46 | 75.01 | -0.60 | 0.95 | 1.50 | 0.42 |
| 3{4} | 0.45 | 310.37 | 5 | 2 | 2 | 0 | 113.88 | 0.04 | -0.09 | -3.27 | 0.51 | 0.38 | 0.48 | 74.02 | -0.54 | 0.99 | 1.73 | 0.53 |
| 3{5} | 1.07 | 324.40 | 5 | 2 | 3 | 0 | 87.94 | 0.25 | 0.56 | -3.95 | 0.84 | 0.39 | 0.41 | 70.00 | -0.35 | 0.37 | 1.28 | 0.23 |
| 3{6} | 1.07 | 324.40 | 5 | 2 | 3 | 0 | 109.05 | 0.10 | 0.01 | -4.16 | 0.74 | 0.36 | 0.34 | 74.32 | -0.58 | 0.56 | 1.25 | 0.48 |
| 3{7} | 1.40 | 337.44 | 4 | 2 | 2 | 0 | 68.08 | 0.33 | 0.79 | -4.16 | 0.85 | 0.12 | 0.68 | 85.87 | -0.44 | 0.63 | 0.94 | -0.01 |
| 3{8} | 1.40 | 337.44 | 4 | 2 | 2 | 0 | 68.46 | 0.33 | 0.82 | -4.11 | 0.91 | 0.08 | 0.72 | 81.83 | -0.53 | 0.53 | 0.77 | 0.01 |
| 3{9} | 1.83 | 338.42 | 5 | 1 | 3 | 0 | 52.34 | 0.15 | 1.07 | -4.60 | 1.08 | 0.26 | 0.72 | 78.22 | -0.45 | 0.45 | 0.69 | -0.33 |
| 3{10} | 1.83 | 338.42 | 5 | 1 | 3 | 0 | 71.81 | 0.15 | 0.39 | -4.14 | 0.93 | 0.24 | 0.67 | 74.36 | -0.71 | 0.57 | 0.81 | -0.09 |
| 4{1} | 2.31 | 308.40 | 4 | 1 | 1 | 0 | 49.01 | 0.12 | 1.24 | -4.39 | 1.10 | 0.25 | 0.86 | 86.48 | -0.54 | 0.83 | 0.44 | -0.31 |
| 4{2} | 2.31 | 308.40 | 4 | 1 | 1 | 0 | 61.58 | 0.12 | 0.75 | -4.01 | 1.09 | 0.19 | 0.92 | 81.13 | -0.67 | 0.66 | 0.65 | -0.03 |
| 4{3} | 0.45 | 310.37 | 5 | 2 | 2 | 0 | 113.67 | 0.04 | 0.13 | -3.50 | 0.59 | 0.35 | 0.46 | 75.01 | -0.60 | 0.95 | 1.50 | 0.42 |
| 4{4} | 0.45 | 310.37 | 5 | 2 | 2 | 0 | 113.88 | 0.04 | -0.09 | -3.27 | 0.51 | 0.38 | 0.48 | 74.02 | -0.54 | 0.99 | 1.73 | 0.53 |
| 4{5} | 1.07 | 324.40 | 5 | 2 | 3 | 0 | 87.94 | 0.25 | 0.56 | -3.95 | 0.84 | 0.39 | 0.41 | 70.00 | -0.35 | 0.37 | 1.28 | 0.23 |
| 4{6} | 1.07 | 324.40 | 5 | 2 | 3 | 0 | 109.05 | 0.10 | 0.01 | -4.16 | 0.74 | 0.36 | 0.34 | 74.32 | -0.58 | 0.56 | 1.25 | 0.48 |
| 4{7} | 1.40 | 337.44 | 4 | 2 | 2 | 0 | 68.08 | 0.33 | 0.79 | -4.16 | 0.85 | 0.12 | 0.68 | 85.87 | -0.44 | 0.63 | 0.94 | -0.01 |
| 4{8} | 1.40 | 337.44 | 4 | 2 | 2 | 0 | 68.46 | 0.33 | 0.82 | -4.11 | 0.91 | 0.08 | 0.72 | 81.83 | -0.53 | 0.53 | 0.77 | 0.01 |
| 4{9} | 1.83 | 338.42 | 5 | 1 | 3 | 0 | 52.34 | 0.15 | 1.07 | -4.60 | 1.08 | 0.26 | 0.72 | 78.22 | -0.45 | 0.45 | 0.69 | -0.33 |
| 4{10} | 1.83 | 338.42 | 5 | 1 | 3 | 0 | 71.81 | 0.15 | 0.39 | -4.14 | 0.93 | 0.24 | 0.67 | 74.36 | -0.71 | 0.57 | 0.81 | -0.09 |
| 5{1} | 3.16 | 336.45 | 4 | 1 | 2 | 0 | 44.72 | 0.45 | 0.91 | -4.50 | 0.91 | 0.14 | 0.66 | 86.30 | -0.51 | 0.14 | 0.55 | -0.40 |
| 5{2} | 3.16 | 336.45 | 4 | 1 | 2 | 0 | 44.75 | 0.45 | 0.85 | -4.62 | 0.96 | 0.15 | 0.62 | 84.26 | -0.47 | -0.03 | 0.58 | -0.35 |
| 5{3} | 1.31 | 338.42 | 5 | 2 | 3 | 0 | 93.34 | 0.04 | 0.06 | -4.14 | 0.31 | 0.34 | 0.32 | 81.54 | -0.33 | 0.58 | 1.50 | 0.01 |
| 5{4} | 1.31 | 338.42 | 5 | 2 | 3 | 0 | 93.33 | 0.04 | 0.01 | -4.04 | 0.30 | 0.33 | 0.34 | 76.43 | -0.35 | 0.66 | 1.46 | -0.04 |
| 5{5} | 1.93 | 352.45 | 5 | 2 | 4 | 0 | 97.12 | 0.79 | 0.15 | -4.35 | 0.46 | 0.30 | 0.17 | 72.36 | -0.22 | 0.49 | 1.78 | 0.11 |
| 5{6} | 1.93 | 352.45 | 5 | 2 | 4 | 0 | 96.63 | 0.79 | 0.19 | -4.14 | 0.54 | 0.38 | 0.26 | 67.72 | -0.23 | 0.60 | 1.74 | 0.22 |
| 5{7} | 2.26 | 365.49 | 4 | 2 | 3 | 0 | 47.44 | 0.33 | 0.82 | -4.71 | 0.68 | 0.09 | 0.49 | 83.36 | -0.41 | 0.18 | 0.66 | -0.56 |
| 5{8} | 2.26 | 365.49 | 4 | 2 | 3 | 0 | 47.39 | 0.33 | 0.91 | -4.89 | 0.72 | 0.04 | 0.48 | 83.16 | -0.40 | 0.01 | 0.60 | -0.61 |
| 5{9} | 2.68 | 366.48 | 5 | 1 | 4 | 0 | 53.95 | 0.13 | 0.74 | -4.78 | 0.74 | 0.11 | 0.50 | 84.42 | -0.49 | 0.36 | 0.90 | -0.42 |
| 5{10} | 2.68 | 366.48 | 5 | 1 | 4 | 0 | 53.76 | 0.13 | 0.64 | -4.67 | 0.76 | 0.14 | 0.52 | 78.18 | -0.39 | 0.24 | 1.14 | -0.43 |
| 6{1} | 3.16 | 336.45 | 4 | 1 | 2 | 0 | 24.09 | 0.45 | 1.12 | -5.07 | 1.35 | -0.01 | 0.88 | 93.64 | -0.82 | 0.19 | 0.10 | -0.32 |
| 6{2} | 3.16 | 336.45 | 4 | 1 | 2 | 0 | 25.72 | 0.45 | 1.13 | -4.58 | 1.41 | 0.23 | 0.89 | 82.01 | -0.71 | 0.01 | 0.41 | -0.24 |
| 6{3} | 1.31 | 338.42 | 5 | 2 | 3 | 0 | 72.90 | 0.28 | 0.02 | -4.11 | 0.67 | 0.27 | 0.52 | 85.13 | -0.61 | 0.80 | 1.44 | 0.14 |
| 6{4} | 1.31 | 338.42 | 5 | 2 | 3 | 0 | 73.01 | 0.28 | -0.06 | -3.96 | 0.71 | 0.26 | 0.47 | 82.31 | -0.55 | 0.66 | 1.67 | 0.43 |
| 6{5} | 1.93 | 352.45 | 5 | 2 | 4 | 0 | 73.24 | 0.10 | 0.19 | -4.46 | 0.77 | 0.25 | 0.45 | 81.75 | -0.37 | 0.61 | 1.89 | 0.22 |
| 6{6} | 1.93 | 352.45 | 5 | 2 | 4 | 0 | 60.10 | 0.13 | 0.30 | -4.12 | 1.10 | 0.29 | 0.62 | 77.94 | -0.63 | 0.24 | 1.43 | 0.16 |
| 6{7} | 2.26 | 365.49 | 4 | 2 | 3 | 0 | 27.03 | 0.33 | 0.96 | -5.08 | 1.05 | 0.00 | 0.63 | 95.81 | -0.49 | -0.16 | 0.83 | -0.17 |
| 6{8} | 2.26 | 365.49 | 4 | 2 | 3 | 0 | 27.10 | 0.33 | 0.90 | -4.92 | 1.07 | 0.02 | 0.67 | 94.22 | -0.50 | 0.03 | 0.81 | -0.14 |
| 6{9} | 2.68 | 366.48 | 5 | 1 | 4 | 0 | 31.99 | 0.15 | 0.83 | -4.91 | 1.09 | 0.05 | 0.73 | 91.30 | -0.63 | 0.33 | 0.98 | -0.34 |
| 6{10} | 2.68 | 366.48 | 5 | 1 | 4 | 0 | 25.16 | 0.17 | 0.91 | -4.85 | 1.35 | 0.11 | 0.84 | 84.46 | -0.61 | -0.13 | 0.68 | -0.28 |
| 7{1} | 3.68 | 350.48 | 4 | 1 | 2 | 0 | 43.13 | 0.45 | 0.73 | -4.54 | 0.96 | 0.07 | 0.70 | 85.66 | -0.58 | 0.13 | 0.67 | -0.48 |
| 7{2} | 3.68 | 350.48 | 4 | 1 | 2 | 0 | 43.15 | 0.45 | 0.74 | -4.49 | 1.01 | 0.09 | 0.66 | 81.46 | -0.53 | 0.00 | 0.67 | -0.37 |
| 7{3} | 1.83 | 352.45 | 5 | 2 | 3 | 0 | 91.74 | 0.79 | -0.11 | -4.19 | 0.38 | 0.25 | 0.38 | 80.29 | -0.40 | 0.55 | 1.62 | -0.05 |
| 7{4} | 1.83 | 352.45 | 5 | 2 | 3 | 0 | 91.73 | 0.79 | -0.15 | -4.11 | 0.38 | 0.24 | 0.41 | 76.13 | -0.42 | 0.62 | 1.57 | -0.11 |
| 7{5} | 2.45 | 366.48 | 5 | 2 | 4 | 0 | 95.53 | 0.79 | 0.04 | -4.32 | 0.51 | 0.25 | 0.22 | 70.80 | -0.24 | 0.52 | 1.84 | 0.04 |
| 7{6} | 2.45 | 366.48 | 5 | 2 | 4 | 0 | 95.04 | 0.79 | -0.14 | -4.19 | 0.55 | 0.31 | 0.27 | 69.69 | -0.36 | 0.64 | 1.79 | 0.21 |
| 7{7} | 2.78 | 379.52 | 4 | 2 | 3 | 0 | 45.84 | 0.33 | 0.65 | -4.76 | 0.71 | 0.01 | 0.54 | 83.56 | -0.46 | 0.15 | 0.83 | -0.63 |
| 7{8} | 2.78 | 379.52 | 4 | 2 | 3 | 0 | 45.79 | 0.33 | 0.73 | -4.97 | 0.74 | -0.03 | 0.53 | 83.74 | -0.45 | -0.03 | 0.76 | -0.67 |
| 7{9} | 3.20 | 380.50 | 5 | 1 | 4 | 0 | 52.35 | 0.13 | 0.61 | -4.70 | 0.77 | 0.09 | 0.53 | 81.86 | -0.49 | 0.42 | 0.98 | -0.40 |
| 7{10} | 3.20 | 380.50 | 5 | 1 | 4 | 0 | 52.17 | 0.13 | 0.48 | -4.63 | 0.83 | 0.14 | 0.57 | 78.23 | -0.49 | 0.39 | 1.07 | -0.32 |
| 8{1} | 3.68 | 350.48 | 4 | 1 | 2 | 0 | 22.49 | 0.45 | 1.26 | -5.37 | 1.41 | -0.06 | 0.87 | 93.74 | -0.71 | 0.00 | 0.29 | -0.61 |
| 8{2} | 3.68 | 350.48 | 4 | 1 | 2 | 0 | 21.93 | 0.45 | 1.05 | -4.86 | 1.40 | 0.15 | 0.89 | 86.02 | -0.68 | -0.01 | 0.55 | -0.33 |

| | | | | | | | | | | | | | | | | | | |
|---|---|---|---|---|---|---|---|---|---|---|---|---|---|---|---|---|---|---|
| 8{3} | 1.83 | 352.45 | 5 | 2 | 3 | 0 | 71.30 | 0.79 | 0.00 | -4.22 | 0.70 | 0.22 | 0.54 | 83.46 | -0.63 | 0.73 | 1.38 | -0.02 |
| 8{4} | 1.83 | 352.45 | 5 | 2 | 3 | 0 | 71.42 | 0.79 | -0.10 | -4.03 | 0.75 | 0.21 | 0.50 | 81.02 | -0.57 | 0.60 | 1.63 | 0.26 |
| 8{5} | 2.45 | 366.48 | 5 | 2 | 4 | 0 | 71.68 | 0.89 | 0.28 | -4.38 | 0.83 | 0.25 | 0.47 | 77.40 | -0.41 | 0.53 | 1.59 | 0.10 |
| 8{6} | 2.45 | 366.48 | 5 | 2 | 4 | 0 | 58.95 | 0.40 | 0.56 | -4.48 | 1.09 | 0.27 | 0.63 | 79.78 | -0.65 | 0.13 | 1.16 | -0.09 |
| 8{7} | 2.78 | 379.52 | 4 | 2 | 3 | 0 | 25.47 | 0.06 | 0.92 | -5.11 | 1.09 | -0.04 | 0.67 | 94.33 | -0.54 | -0.25 | 0.82 | -0.29 |
| 8{8} | 2.78 | 379.52 | 4 | 2 | 3 | 0 | 25.55 | 0.06 | 0.86 | -4.97 | 1.10 | -0.02 | 0.70 | 92.80 | -0.55 | -0.08 | 0.80 | -0.25 |
| 8{9} | 3.20 | 380.50 | 5 | 1 | 4 | 0 | 30.44 | 0.17 | 0.93 | -4.81 | 1.14 | 0.07 | 0.73 | 86.24 | -0.67 | 0.26 | 0.70 | -0.39 |
| 8{10} | 3.20 | 380.50 | 5 | 1 | 4 | 0 | 23.66 | 0.16 | 1.00 | -4.67 | 1.24 | 0.18 | 0.81 | 80.85 | -0.65 | -0.07 | 0.64 | -0.37 |
| **Averages** | 1.97 | 341.34 | 5 | 2 | 3 | 0 | 73.38 | 0.29 | 0.49 | -4.29 | 0.79 | 0.22 | 0.53 | 78.49 | -0.46 | 0.44 | 1.10 | -0.06 |

## References


1. Pangborg, A. B.; Giardello, M. A.; Grubbs, R. H.; Rosen, R. K.; Timmers, F. J. *Organometallics* **1996,** *15,* 1518–1520. doi:10.1021/om9503712

2. *SYBYL 8.0,* The Tripos Associates: St. Louis, MO, 2008.
3. Lipinski, C. A.; Lombardo, F.; Dominy, B. W.; Feeney, P. J. *Adv. Drug Delivery Rev.* **1997,** *23,* 3–25. doi:10.1016/S0169-409X(96)00423-1

4. *Concord 8.0,* The Tripos Associates, St. Louis MO, 2008.

5. Cruciani, G.; Meniconi, M.; Carosati, E.; Zamora, I.; Mannhold, R. VOLSURF: A Tool for Drug ADME-Properties Prediction. In *Methods and Principles in Medicinal Chemistry;* van de Waterbeemd, H.; Lennernäs, H.; Artursson, P.; Eds.; Wiley-VCH Verlag GmbH & Co.: Weinheim, 2003.

6. Pearlman, R. S.; Smith, K. M. *J. Chem. Inf. Comput. Sci.* **1999,** *39,* 28–35. doi:10.1021/ci980137x
